# Supplementary material for: Unconventional mechanical and thermal behaviours of MOF CALF-20
Source: Nat Commun. 2024 Apr 16;15:3251. doi: 10.1038/s41467-024-47695-6 (PMC11021538; doi:10.1038/s41467-024-47695-6)
Supplement: Supplementary file 1 — Supplementary Information [file 41467_2024_47695_MOESM1_ESM.pdf]

Supplementary Information

for

Unconventional mechanical and thermal behaviours of MOF CALF-20

Dong Fan,[1,2] Supriyo Naskar,[1] Guillaume Maurin[1,*]

1 ICGM, Univ. Montpellier, CNRS, ENSCM, Montpellier, 34095, France

2 School of Materials Science and Engineering, Chongqing Jiaotong University, Chongqing 400074, PR China

*Corresponding author: guillaume.maurin1@umontpellier.fr

Index **Pages**

1	**Supplementary Figures** ······································	2-40
2	**Supplementary Tables**··	41-48
3	**Supplementary Notes**··	49-59
4	**Supplementary References**··································	60-63

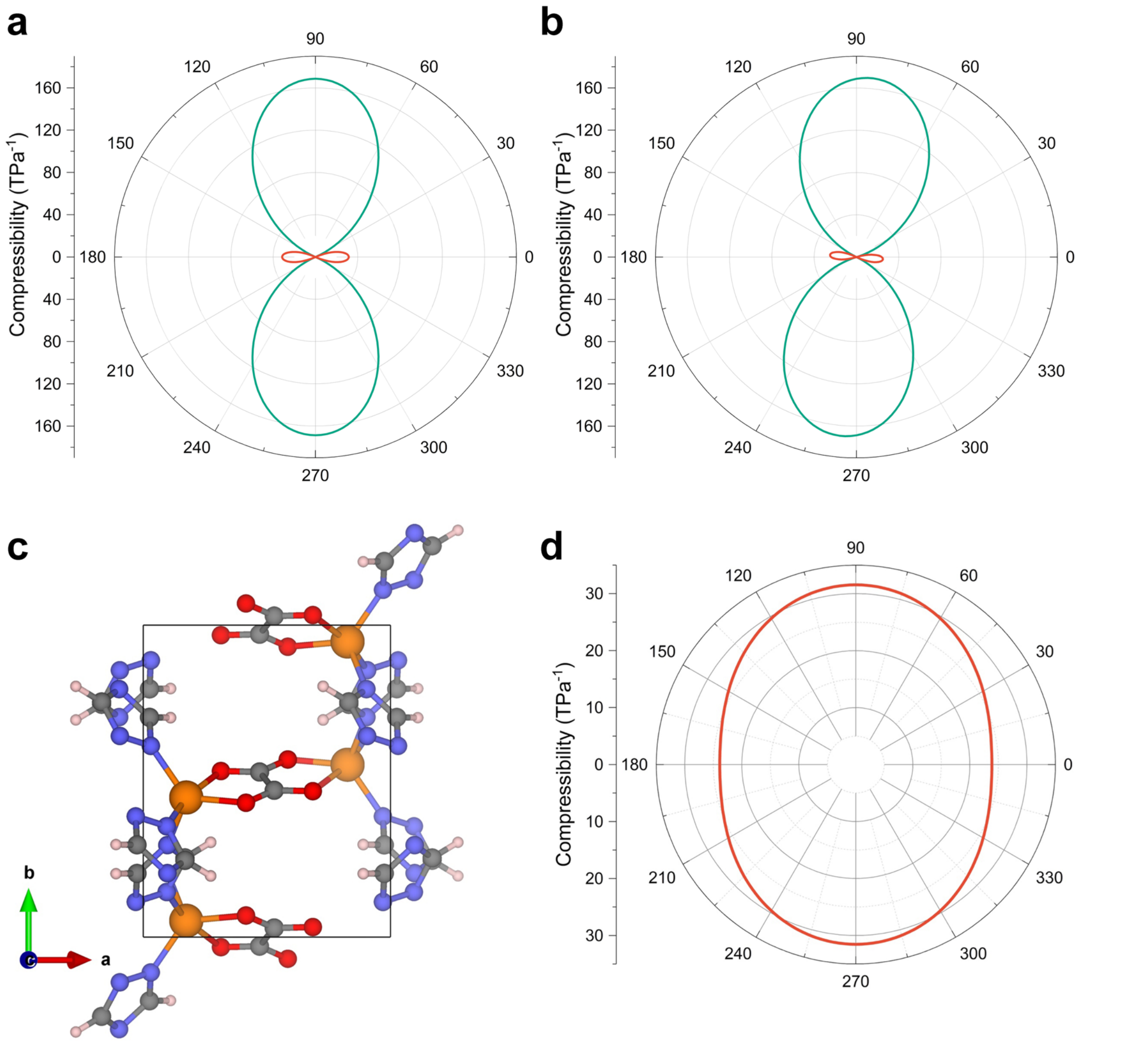

Supplementary Fig. 1 | The elastic constant derived spatial dependence of the linear compressibility of CALF-20 in (**a**) ***bc*** and (**b**) ***ac*** plane, respectively (**c**) Top view of CALF-20 structure, and the corresponding (**d**) compressibility in ***ab*** plane. The dark-green and red line indicate positive and negative contributions, respectively. Color code: Zn, orange; N, blue; O, red; C, grey; H, white.

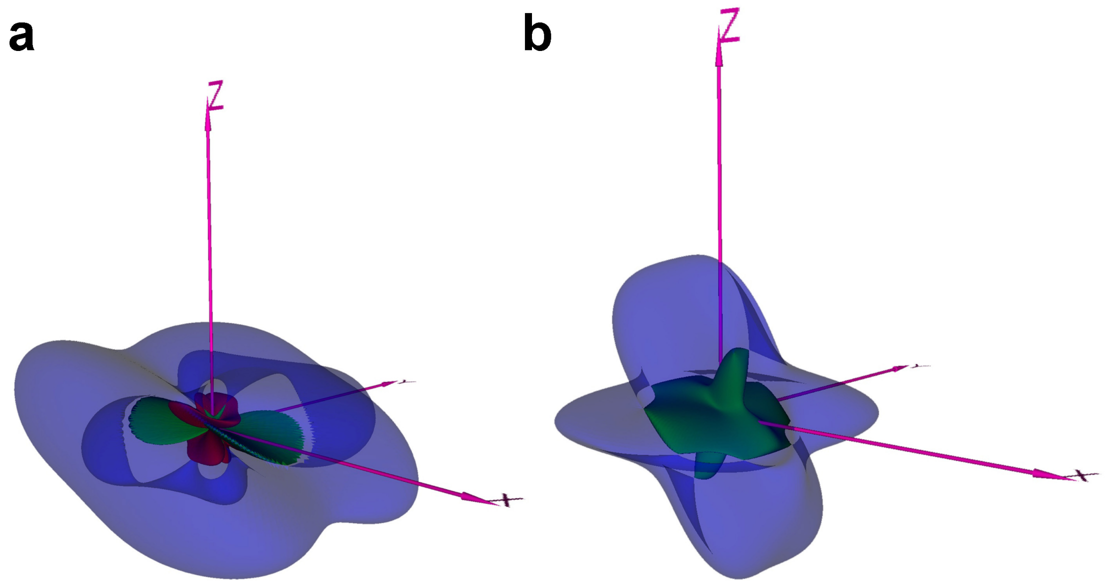

Supplementary Fig. 2 | Three dimensional representations of the (**a**) Poisson's ratio, and (**b**) shear modulus for CALF-20, respectively. The maximum values are showed in blue, while green and red surfaces correspond to positive and negative contributions, respectively.

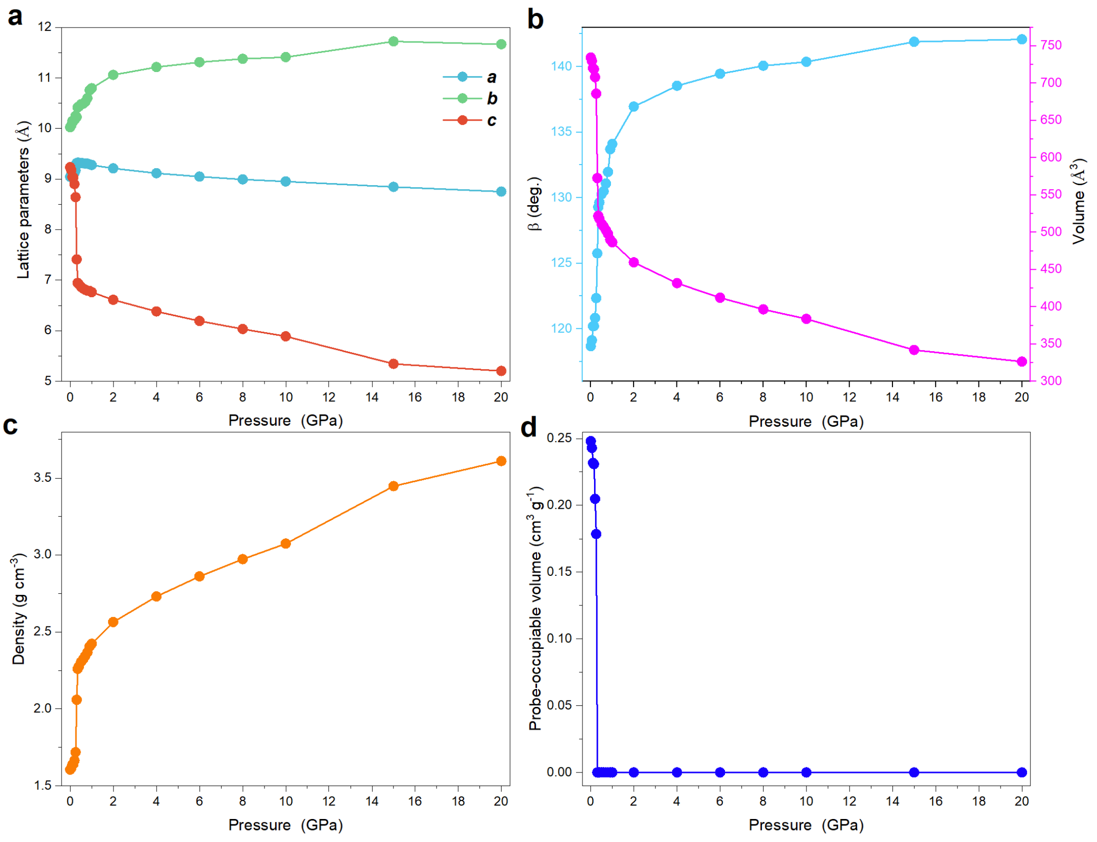

Supplementary Fig. 3 | (a, b) Unit cell parameters changes (0-20 GPa) of the CALF-20 during hydrostatic compression based on PBE-D3 functional calculations. The **(d)** density and **(e)** probe-occupiable volume accessible to a probe of radius of 1.2 Å in CALF-20, respectively. Note that starting from 0.3 GPa pressure, accessible volume becomes 0.

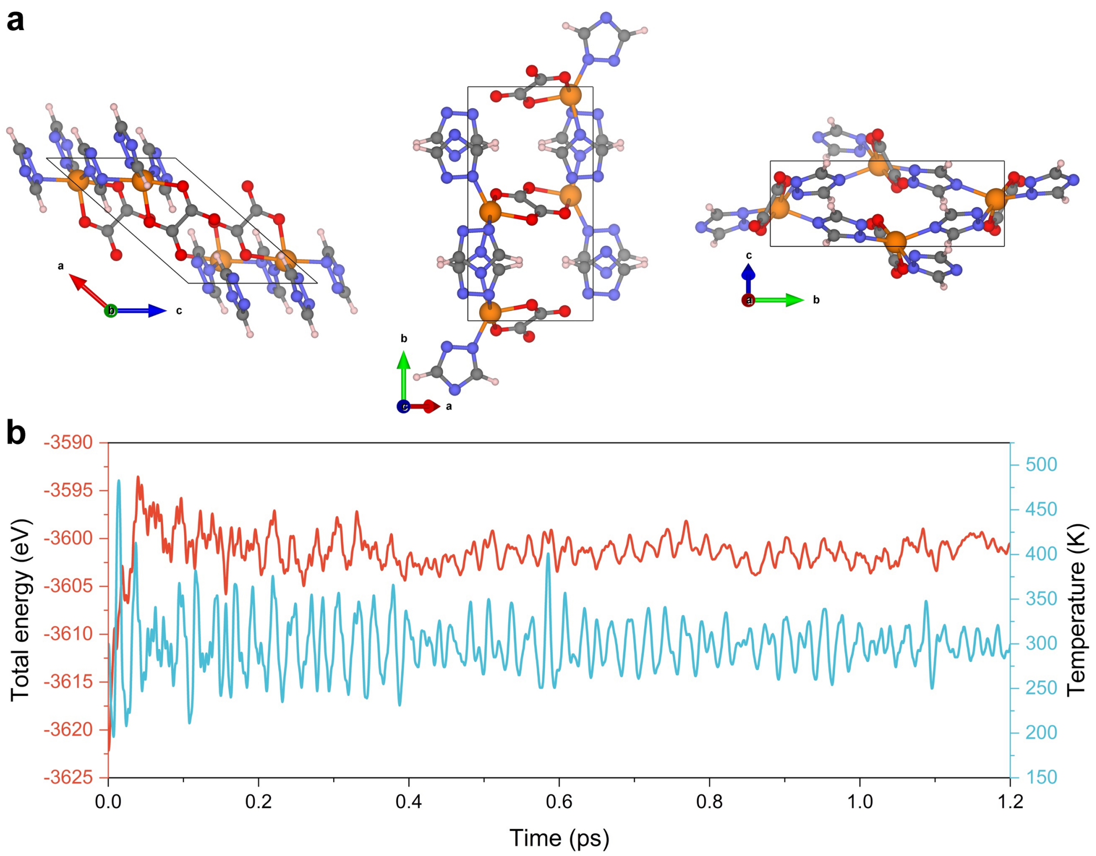

Supplementary Fig. 4 | (**a**)The high-pressure CALF-20 phase structure extracted from the pristine CALF-20 structure at 15.0 GPa pressure and then depressurized for the high-accuracy DFT structure optimization (including atom position and lattice parameter optimizations). According to our calculations, the high-pressure phase is higher than the pristine CALF-20 with 43.05 meV per atom. (**b**) AIMD simulated total energy and temperature profiles of the meta-stable CALF-20 supercell with simulation time. Where the simulation time step is set as 0.5 fs (*NVT* ensemble at room temperature).

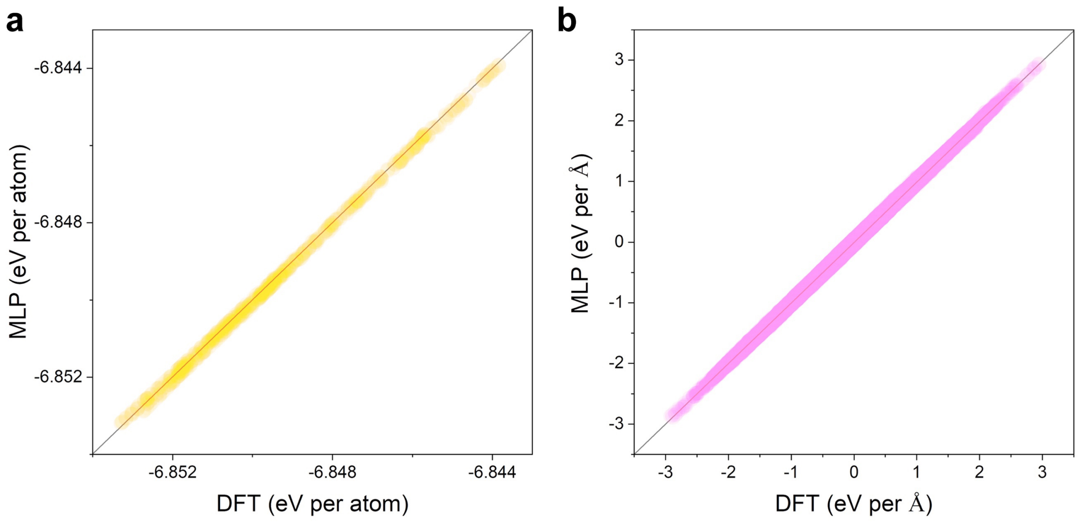

Supplementary Fig. 5 | Machine learning potential (MLP) derived (**a**) energies and (**b**) forces *vs* the corresponding density functional theory (DFT) values in the potential testing.

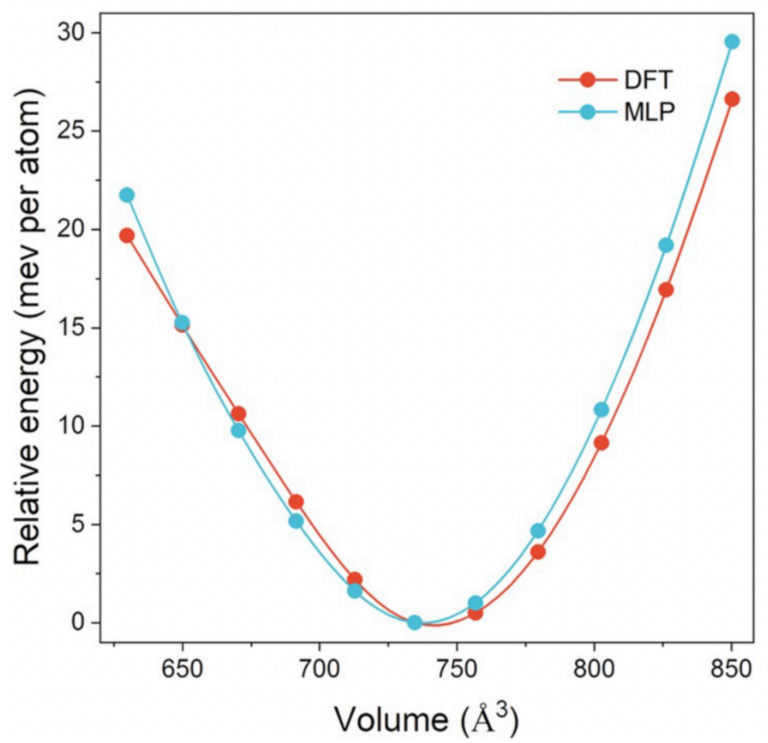

Supplementary Fig. 6 | Volume-energy curves for CALF-20 using DFT and MLP calculations.

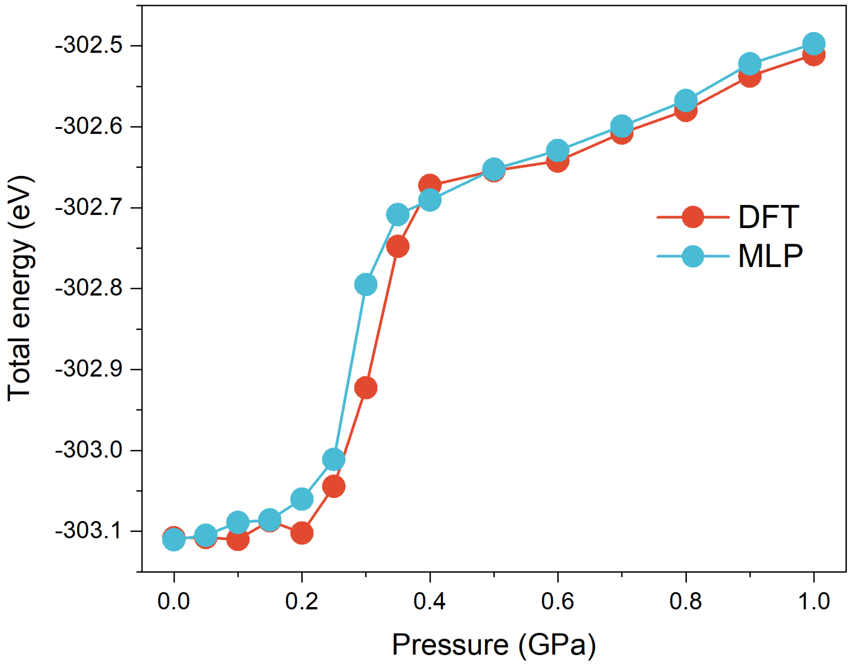

Supplementary Fig. 7 | The total energy of the CALF-20 (unit cell) driven by DFT and MLP under different pressures, respectively.

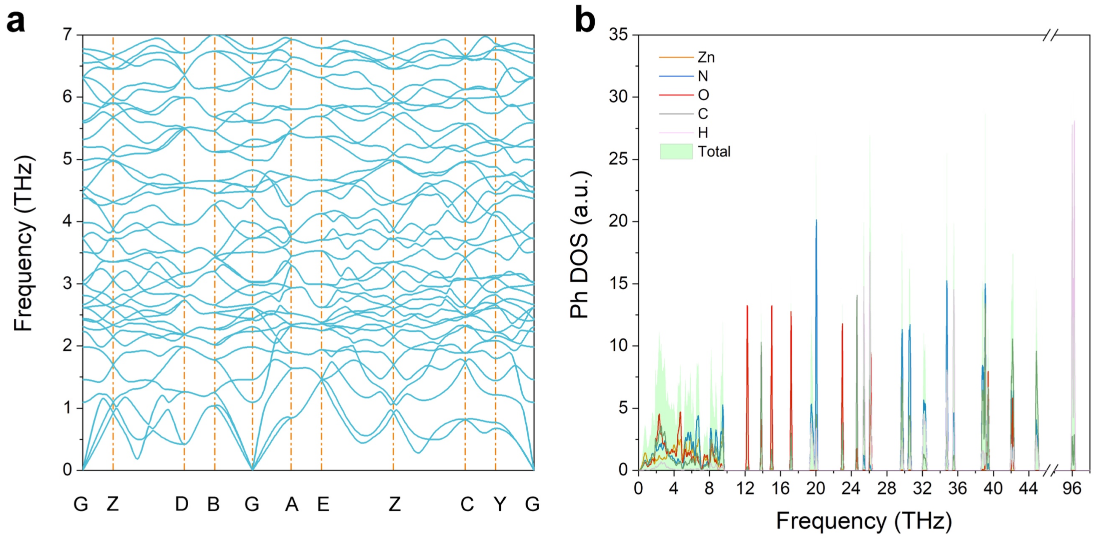

Supplementary Fig. 8 | (a) Phonon spectrum (only below 7 THz curves are displayed for clarity) and (**b**) phonon density of states (Ph DOS) of CALF-20 predicted by the MLP.

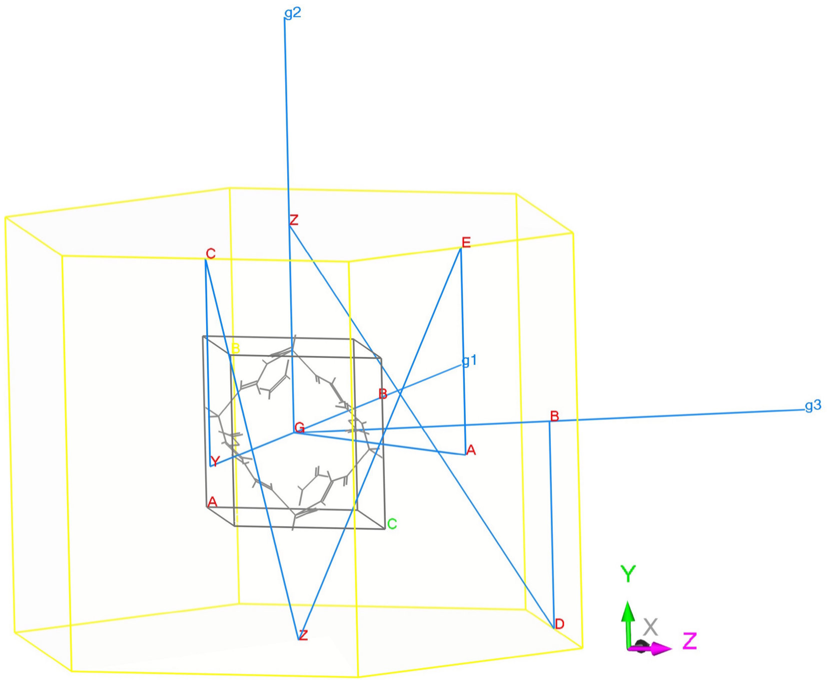

Supplementary Fig. 9 | Schematic diagram of the Brillouin zone path of the CALF-20 structure. The corresponding high symmetry point (red) is sampled in the phonon dispersion spectrum calculations.

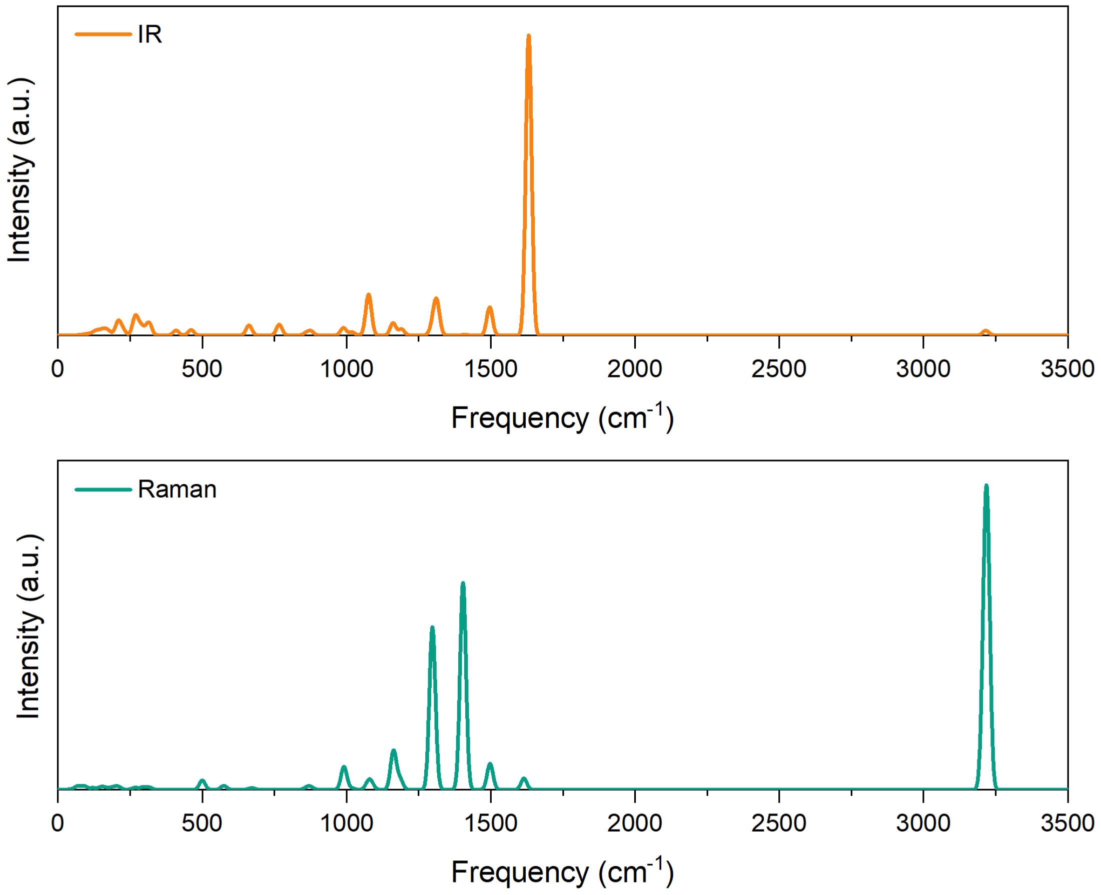

Supplementary Fig. 10 | Simulated IR (top) and Raman (bottom) images of the CALF-20 structure.

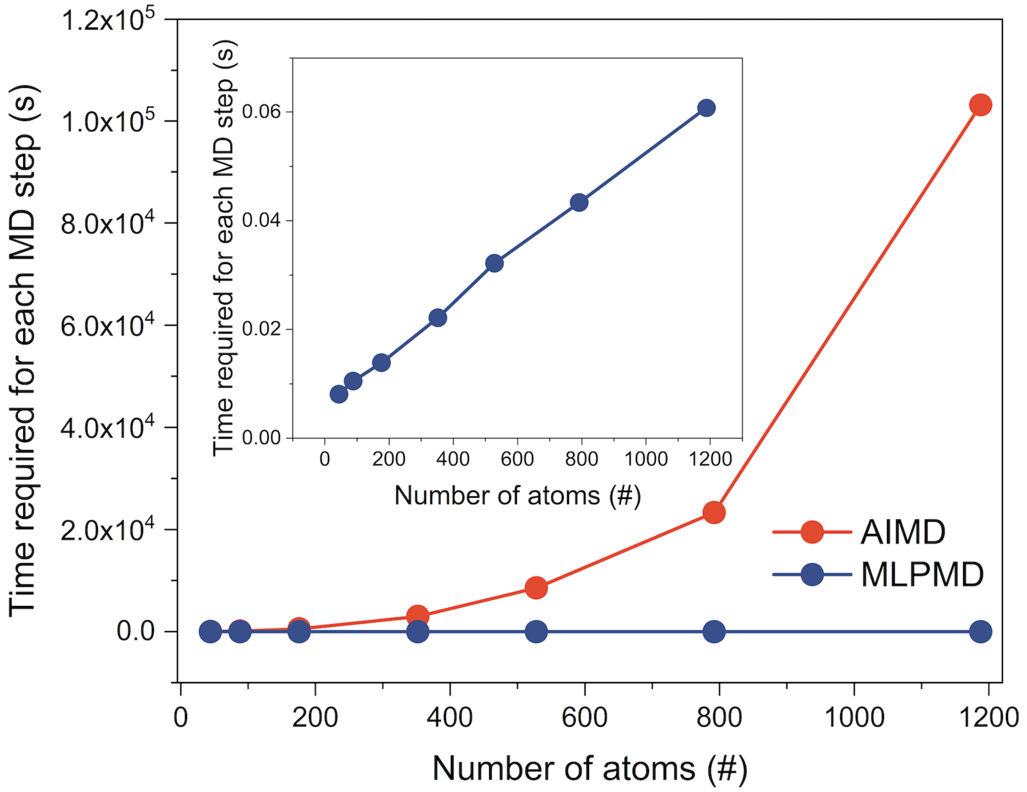

Supplementary Fig. 11 | The CPU time comparison between AIMD and MLP-MD simulations. For each system, the same CPU (96-cores) was used to compute the average time per MD time step in seconds.

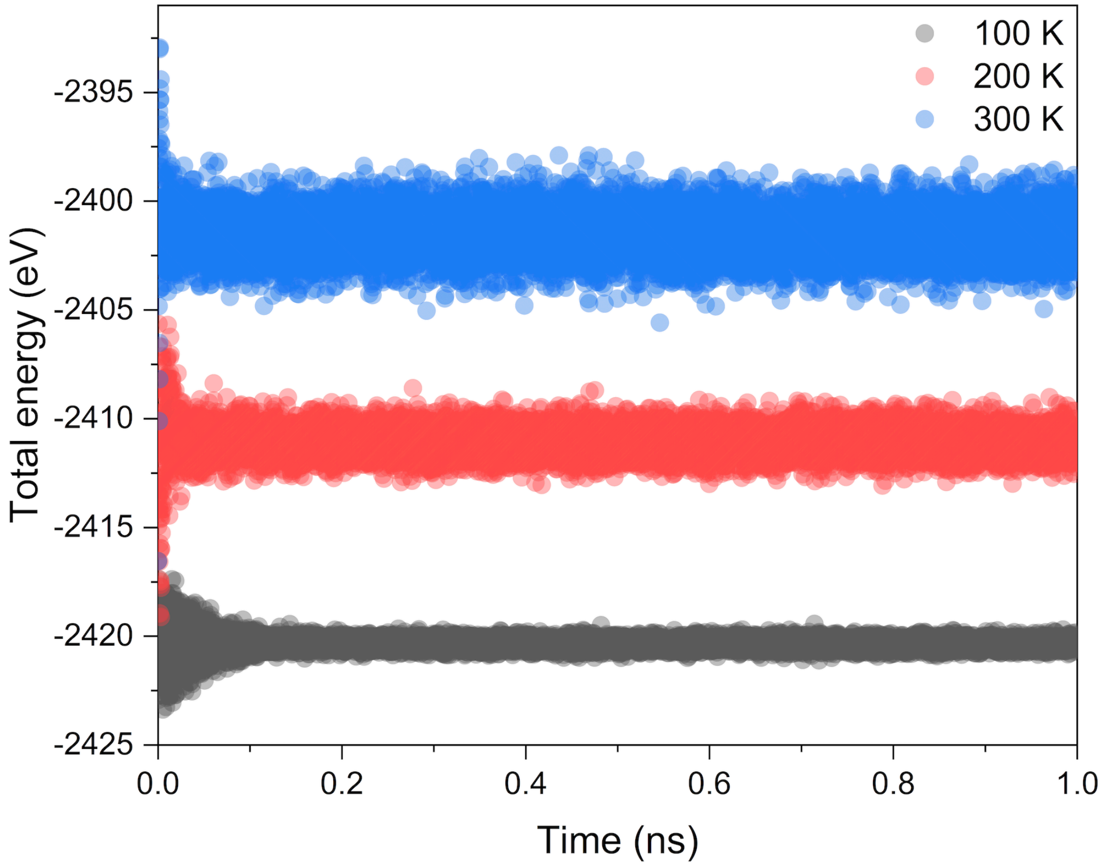

Supplementary Fig. 12 | Energy fluctuations of the CALF-20 (2×2×2 supercell) during the MLP-MD simulations at different temperatures

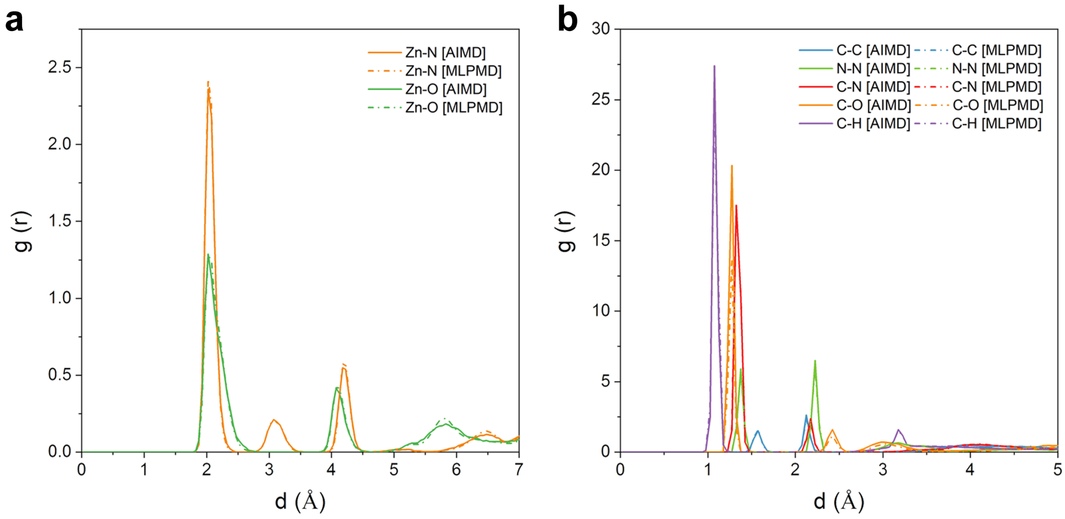

Supplementary Fig. 13 | (a, b) Statistical Radial Distribution Functions (RDFs) calculated for the pairs of CALF-20 at 300 K by using MLP and AIMD simulations, respectively.

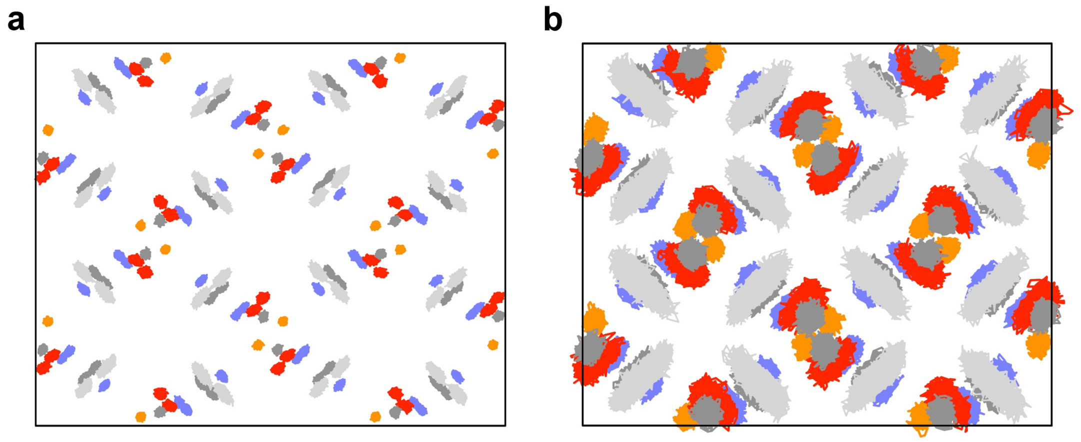

Supplementary Fig. 14 | Atomic trajectories from the MLP-MD simulations at (**a**) 100 K and (**b**) 500 K of CALF-20 (2×2×2 supercell, view along [100] direction). Color code: Zn, orange; N, blue; O, red; C, grey; H, light-grey.

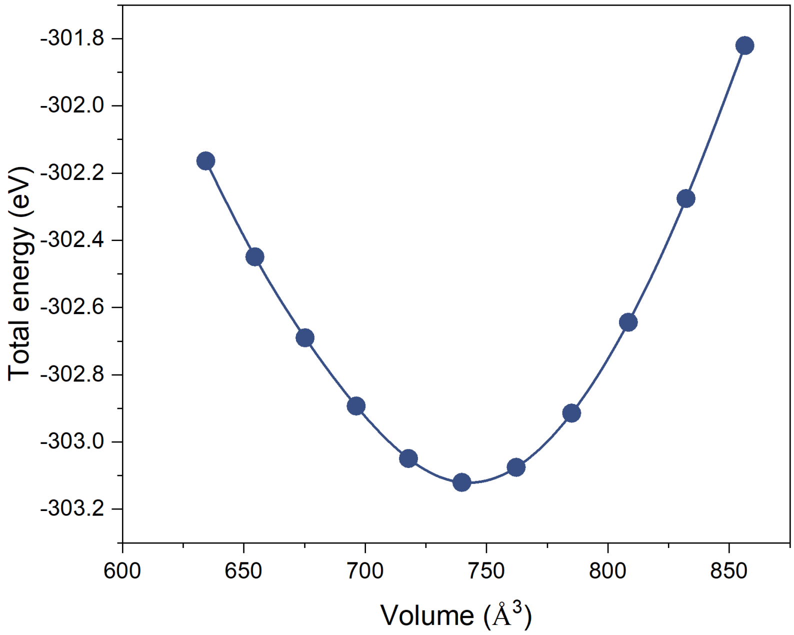

Supplementary Fig. 15 | Volume *vs* energy plot for CALF-20 from MLP simulation.

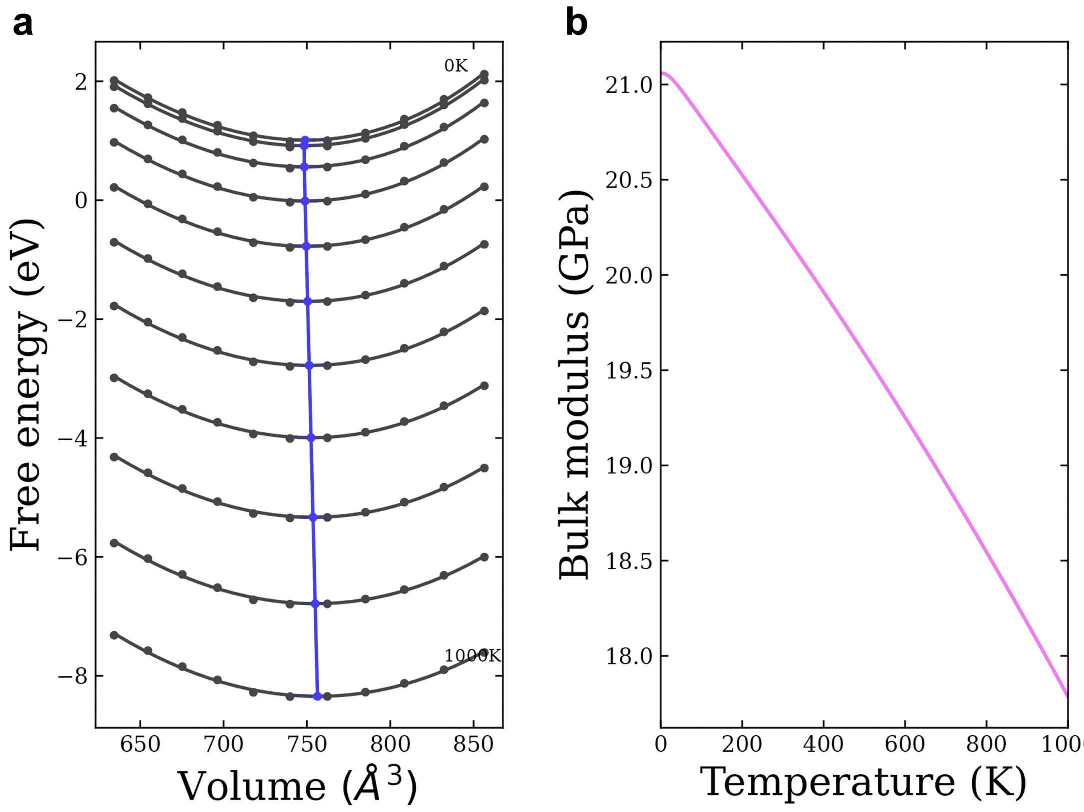

Supplementary Fig. 16 | (**a**) Helmholtz free energies and (**b**) bulk modulus of the CALF-20 *versus* volume at the temperature scale from 0 K to 1000 K with 100 K steps are shown as filled circles (black) and the values are fitted by the solid curves. The cross symbols (blue) indicate the energy bottoms of each curve and the equilibrium volumes. (**b**) Evolution of bulk modulus with temperature from fitting of volume-energy data.

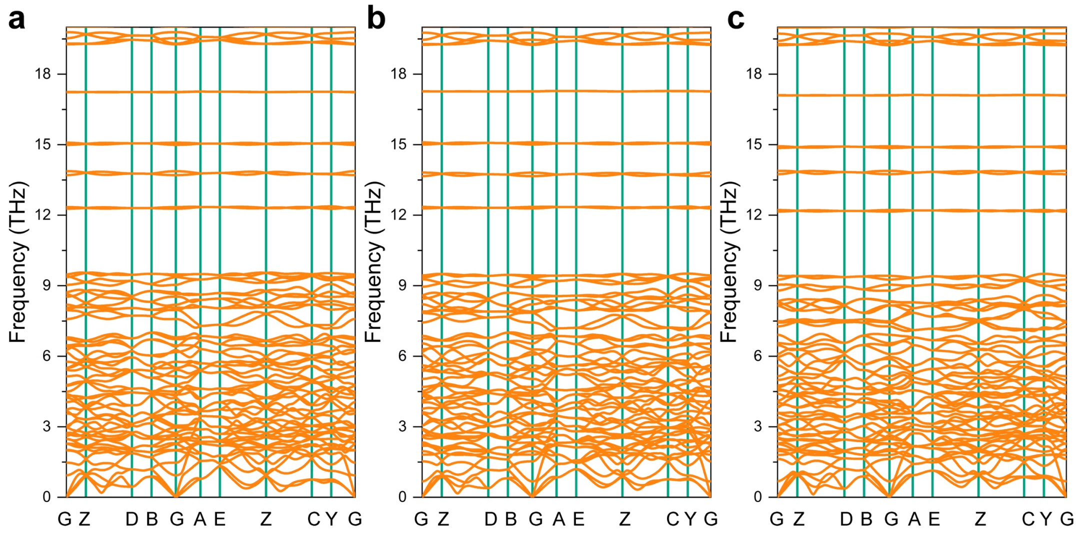

Supplementary Fig. 17 | Calculated phonon spectrum of the CALF-20 structure under (**a**) 2.00%, (**b**) 4.07%, and (**c**) 8.25% deformation by applying tensile strain along the [100] direction based on the MLP. Only below 20 THz curves are displayed for clarity.

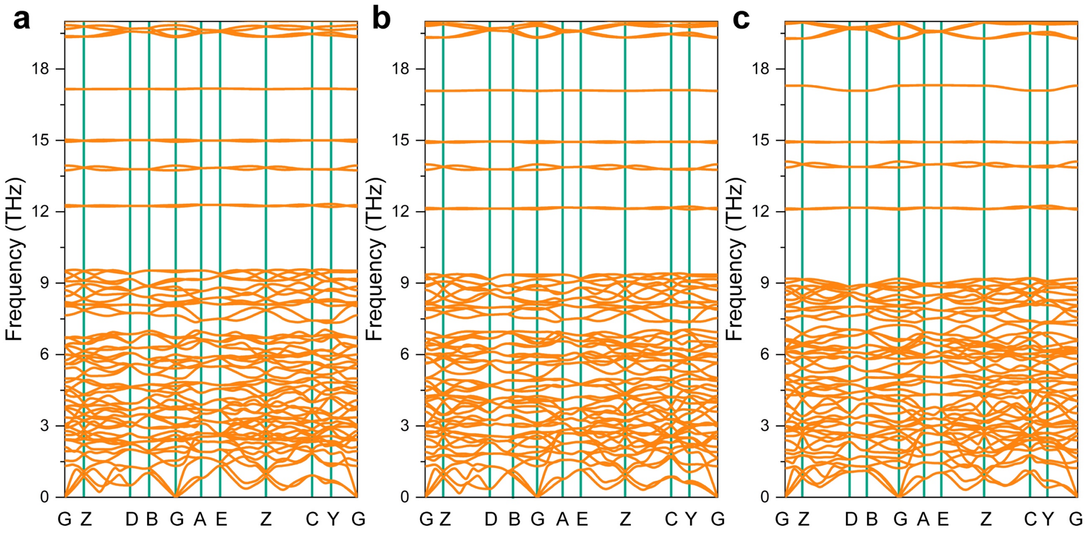

Supplementary Fig. 18 | Calculated phonon spectrum of the CALF-20 structure under (**a**) 5.10%, (**b**) 10.46%, and (**c**) 14.95% deformation by applying tensile strain along the [010] direction based on the MLP. Only below 20 THz curves are displayed for clarity.

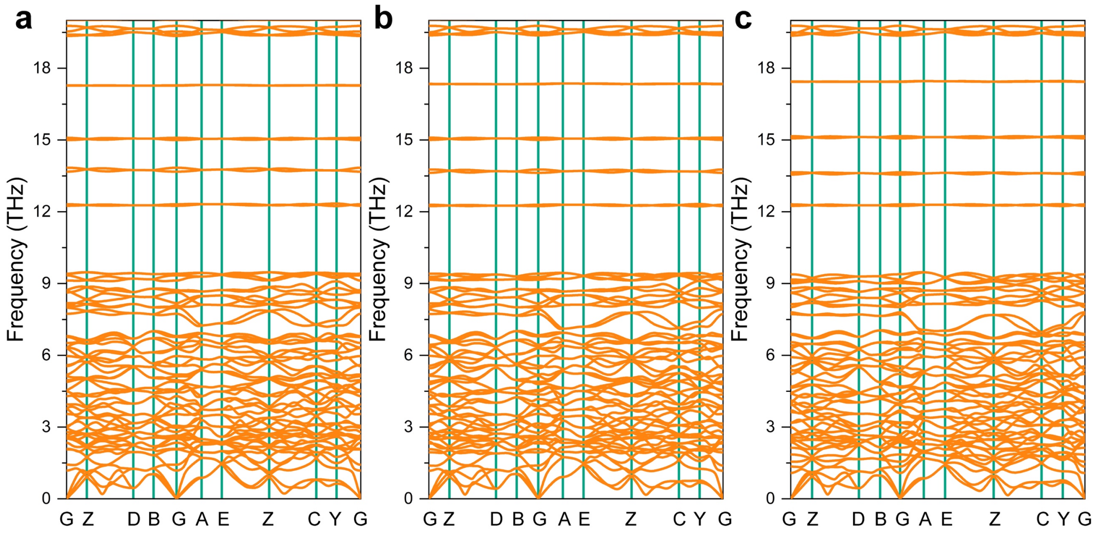

Supplementary Fig. 19 | Calculated phonon spectrum of the CALF-20 structure under (**a**) 5.10%, (**b**) 10.46%, and (**c**) 14.95% deformation by applying tensile strain along the [001] direction based on the MLP. Only below 20 THz curves are displayed for clarity.

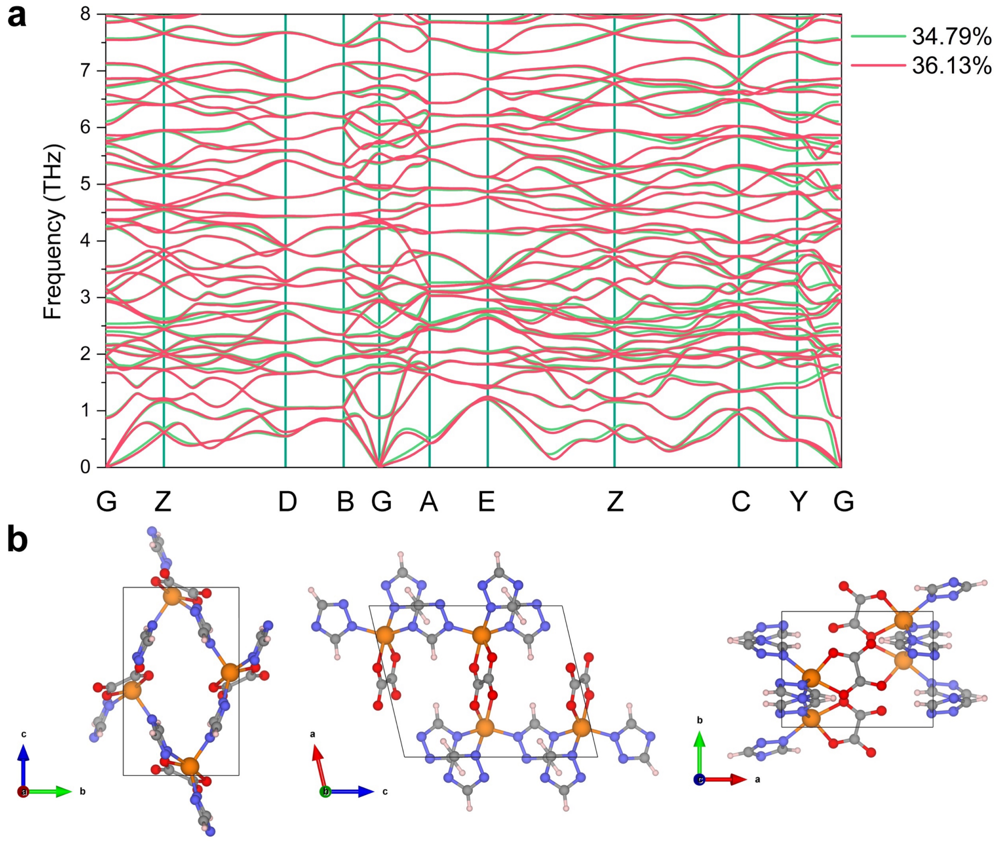

Supplementary Fig. 20 | (a) The phonon dispersion spectrum for extreme strains (34.79% and 36.13%) applied to the CALF-20 structure along the [001] direction. Only below 8 THz curves are displayed for clarity. (**b**) The atomic structure of the corresponding CALF-20 under 36.13% strain along the [001] direction.

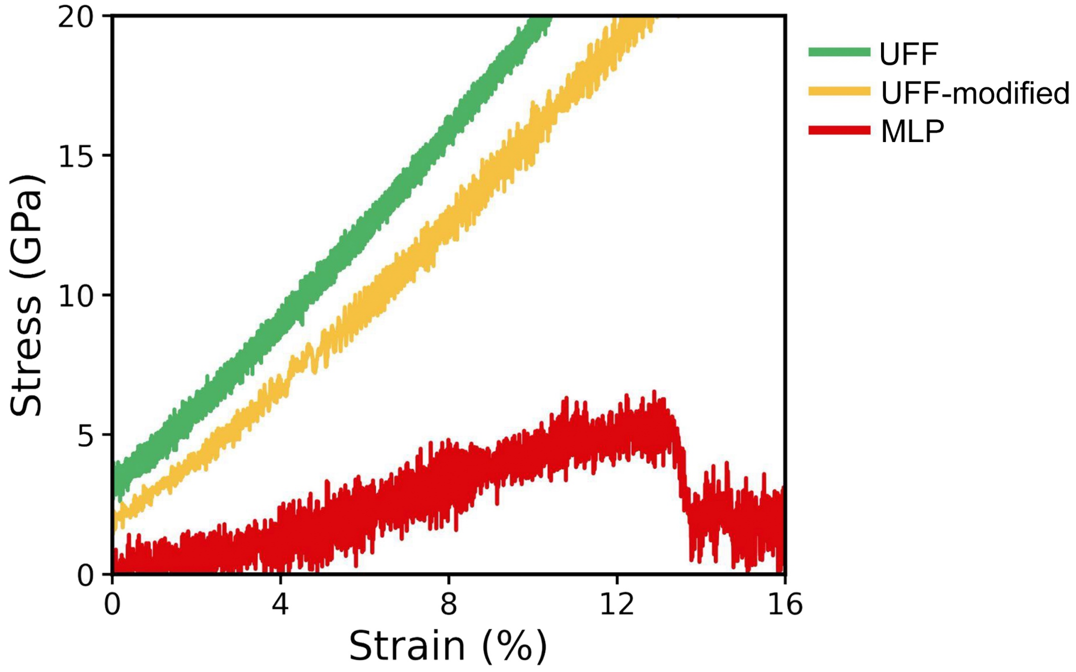

Supplementary Fig. 21 | Stress-strain curves of the CALF-20 structure with tensile strain applied along the [100] direction at room temperature, derived from UFF and MLP-MD simulations. The simulations employed a 2×2×2 (3×3×3) supercell for MLP-MD (UFF and UFF-modified) and were performed in the *NPT* ensemble at 1 bar condition

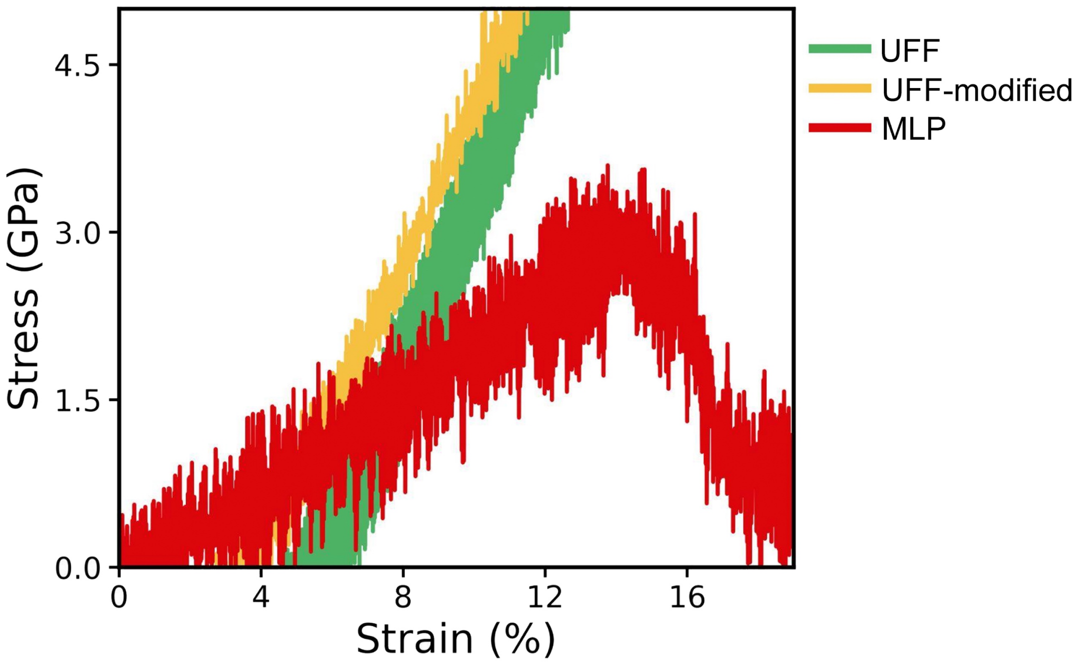

Supplementary Fig. 22 | Stress-strain curves of the CALF-20 structure with tensile strain applied along the [010] direction at room temperature, derived from UFF and MLP-MD simulations. The simulations employed a 2×2×2 (3×3×3) supercell for MLP-MD (UFF and UFF-modified) and were performed in the *NPT* ensemble at 1 bar condition

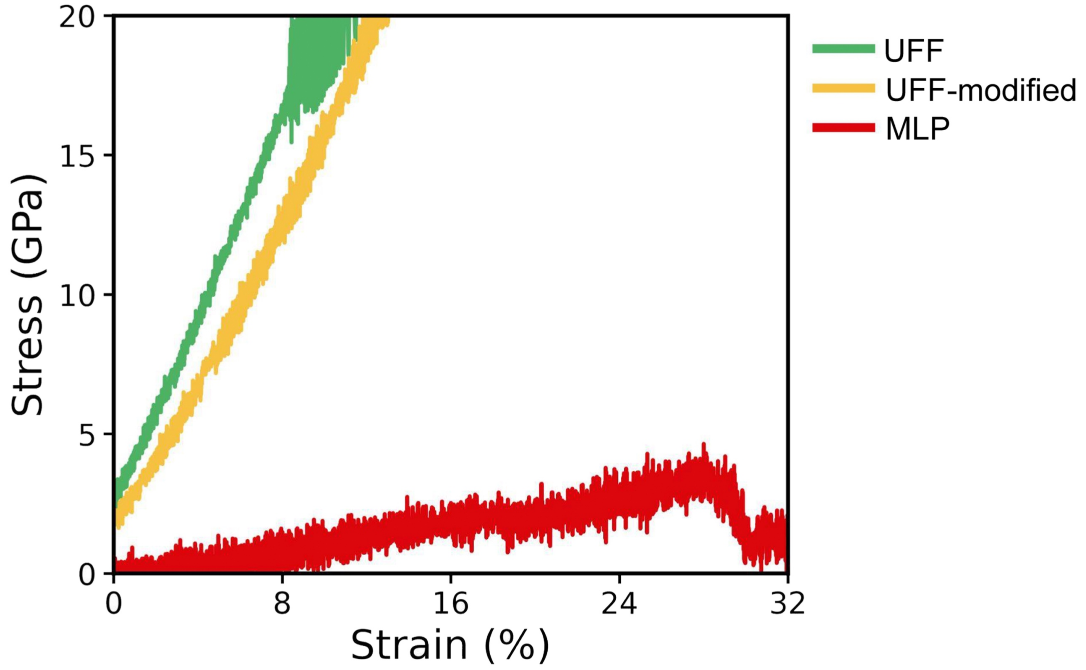

Supplementary Fig. 23 | Stress-strain curves of the CALF-20 structure with tensile strain applied along the [001] direction at room temperature, derived from UFF and MLP-MD simulations. The simulations employed a 2×2×2 (3×3×3) supercell for MLP-MD (UFF and UFF-modified) and were performed in the *NPT* ensemble at 1 bar condition.

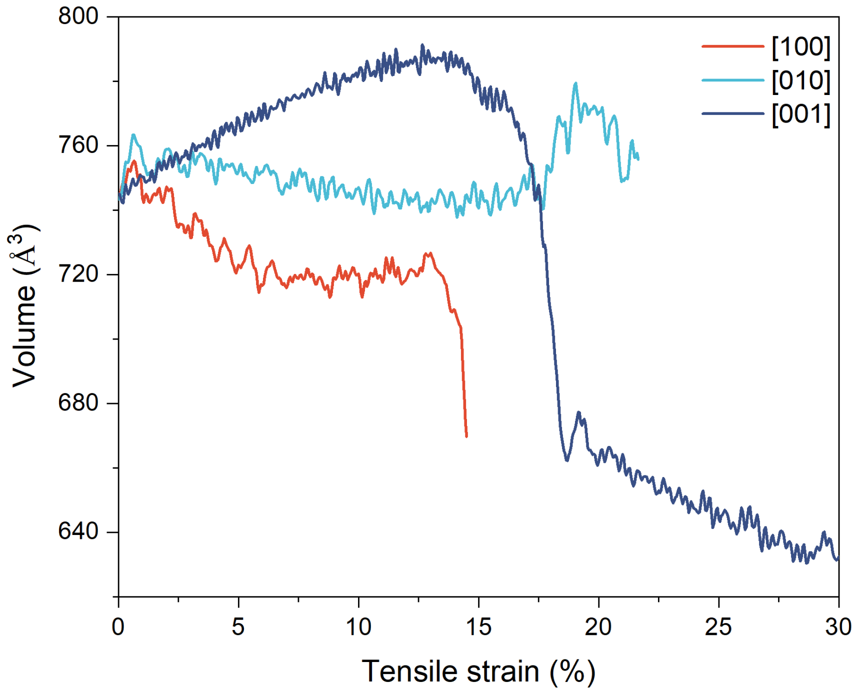

Supplementary Fig. 24 | Volumetric changes of the CALF-20 structure with applied tensile strain along the different directions at 200 K from MLP-MD simulations (Data at room temperature given in the main text, Fig. 3f).

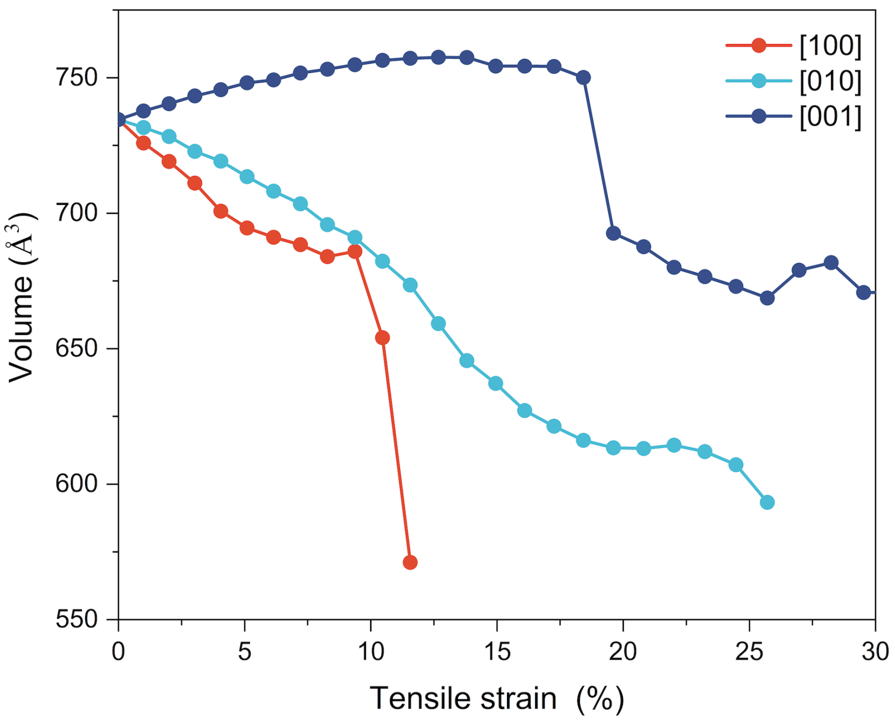

Supplementary Fig. 25 | Volumetric changes of CALF-20 structure with applied tensile strain along the different directions at 0 K from DFT calculations.

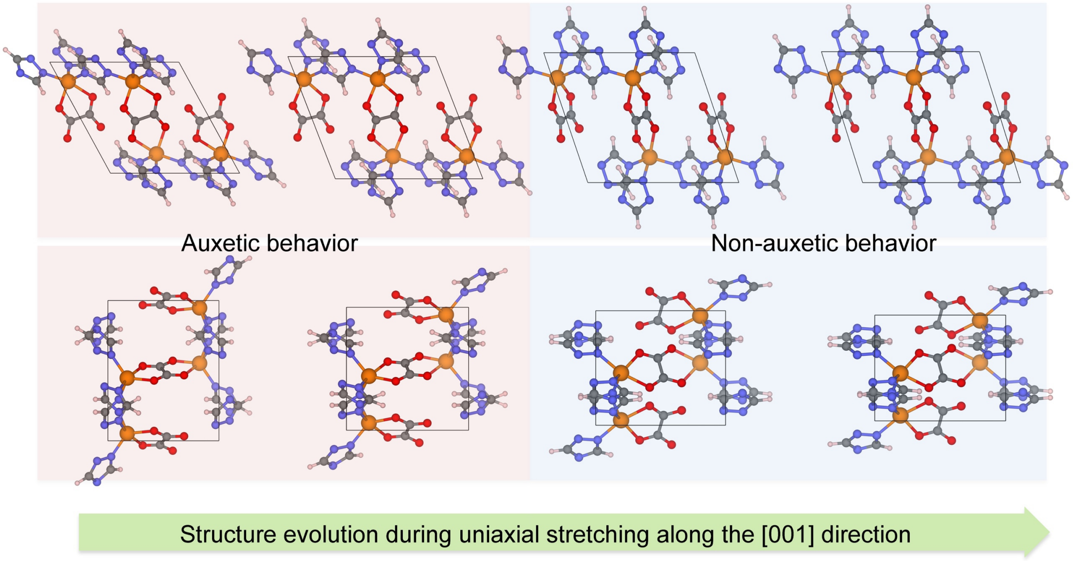

Supplementary Fig. 26 | Schematic representation of the structural changes of CALF-20 when tensile strain is applied along the [001] direction, complementary to Fig. 4g in the main text (viewed along different directions).

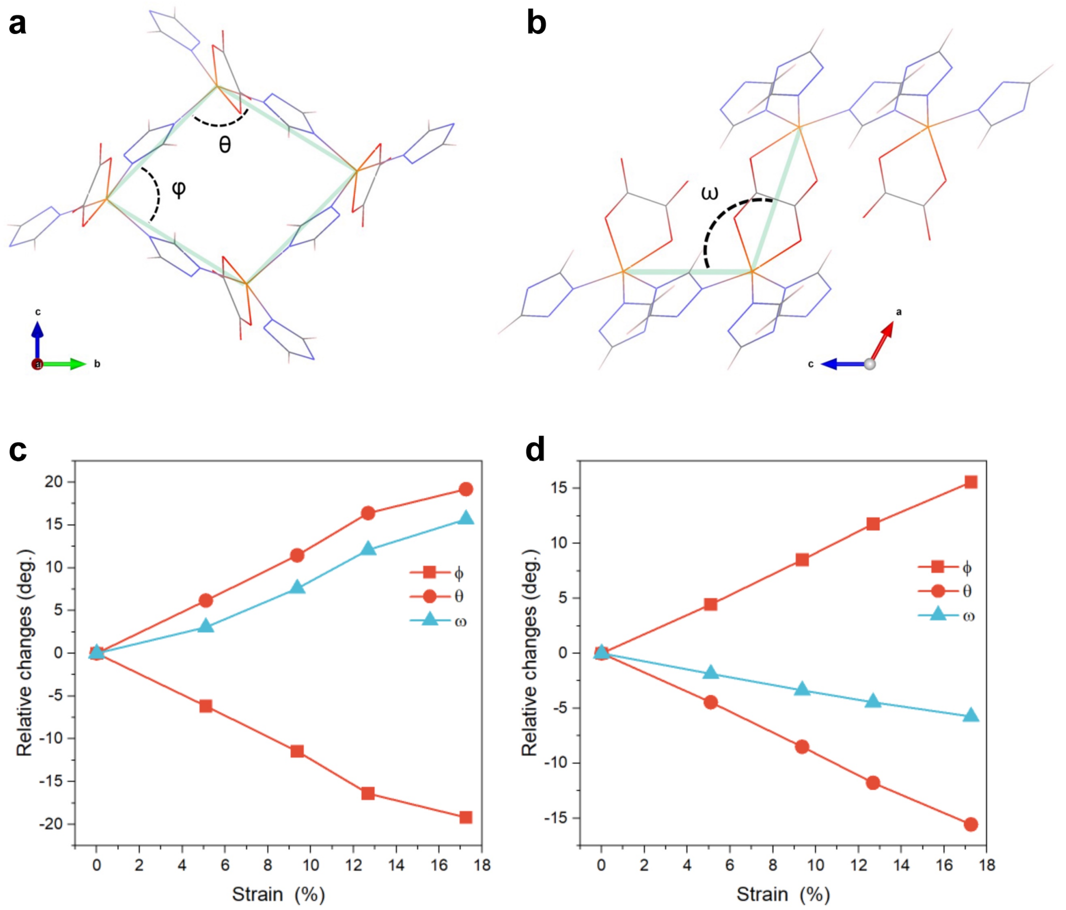

Supplementary Fig. 27 | (**a**) The scheme of the CALF-20 along the ***bc*** and ***ac*** planes, where the angle ϕ and θ denote the angle between adjacent triazolate ligands in the lozenge lattice of CALF-20, and the angle ω represents the angle between the triazolate ligand and oxalate ligand. Angle changes (ϕ, θ, and ω) for the applied tension strain along the (**c**) [010] and (**d**) [001] direction, respectively.

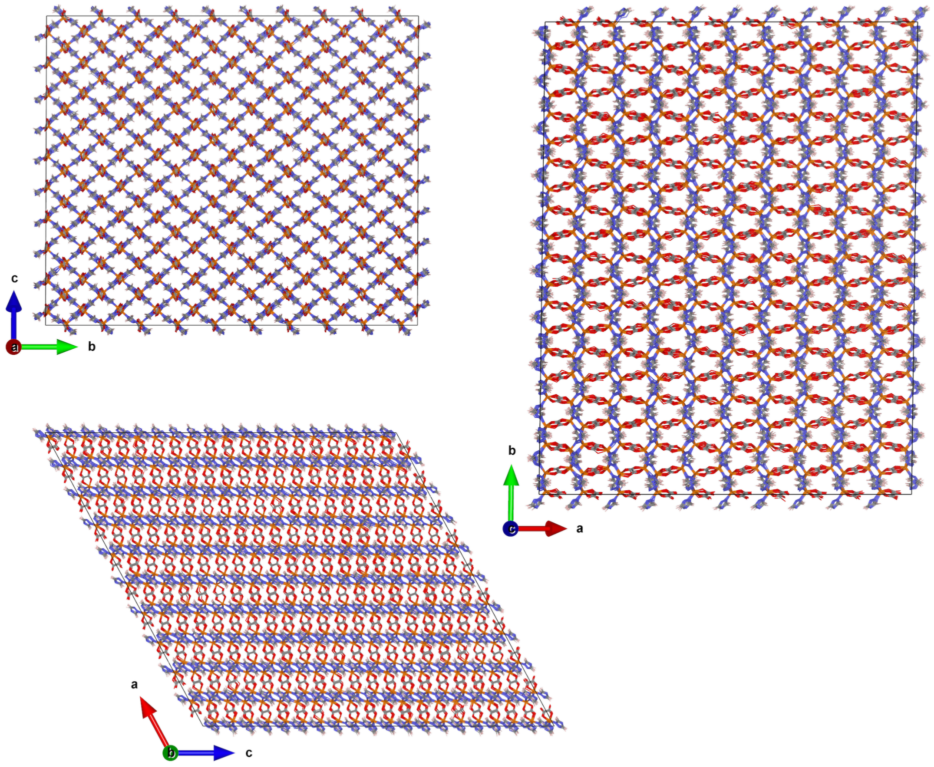

Supplementary Fig. 28 | Snapshot of the CALF-20 supercell structure (different views) with 44,000 atoms after 100 ps simulation using MLP-MD simulations at room temperature (298.15 K, *NVT* ensemble). Color code: Zn, orange; N, blue; O, red; C, grey; H, white.

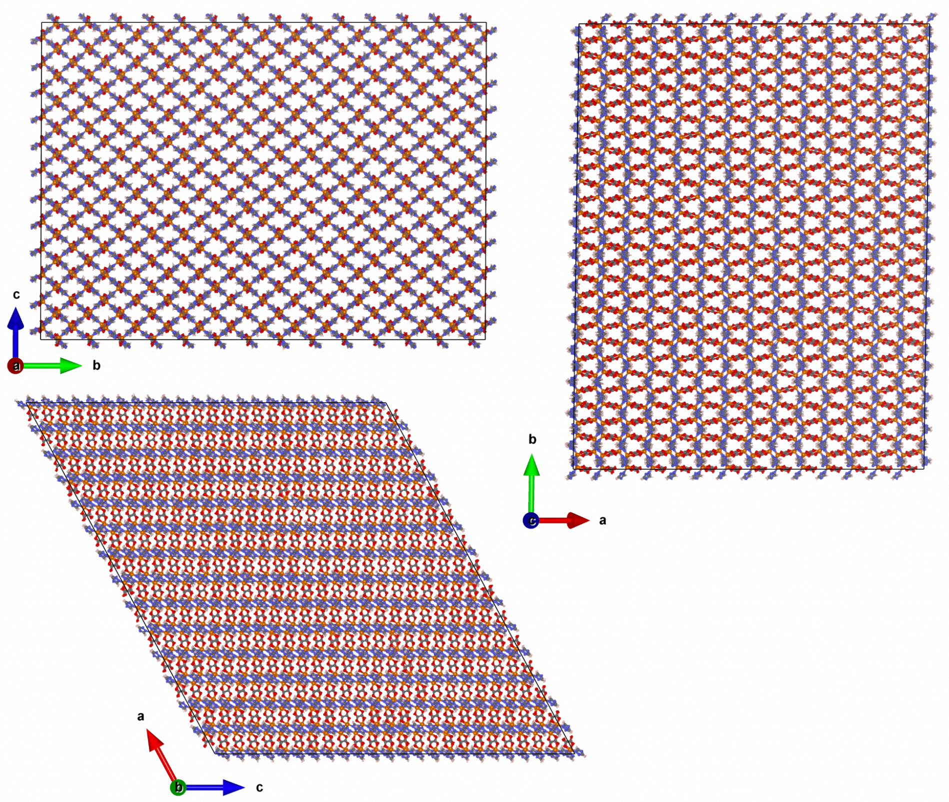

Supplementary Fig. 29 | Snapshot of the CALF-20 supercell structure (different views) with 134,400 atoms after 100 ps simulation using MLP-MD simulations at room temperature (298.15 K, *NVT* ensemble). Color code: Zn, orange; N, blue; O, red; C, grey; H, white.

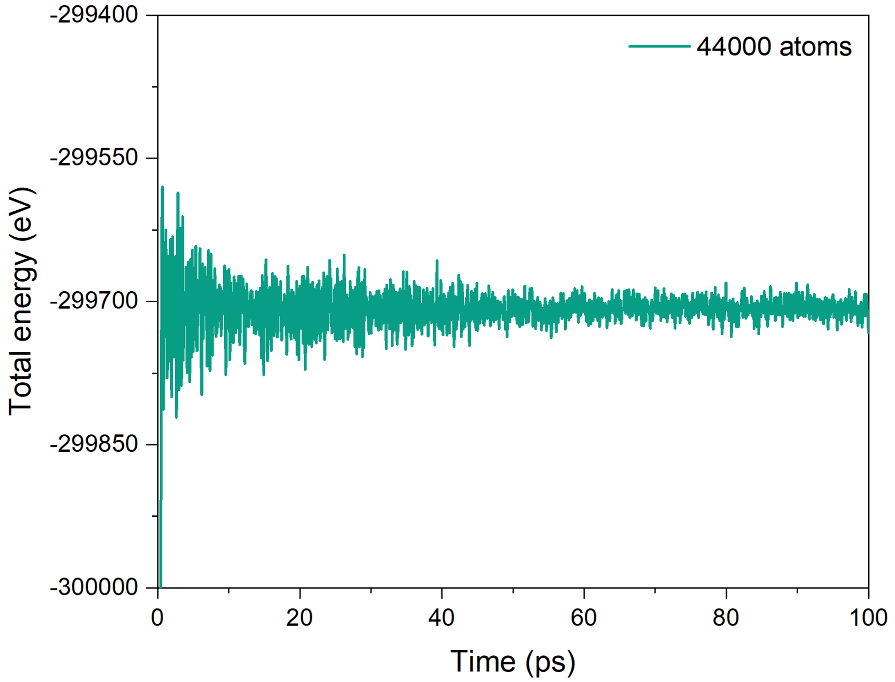

Supplementary Fig. 30 | Evolution of the total energy obtained by MLP-MD simulations for CALF-20 supercell (44,000 atoms) MLP-MD simulations in the *NVT* ensemble (T=298.15 K) and a timestep of 0.5 fs.

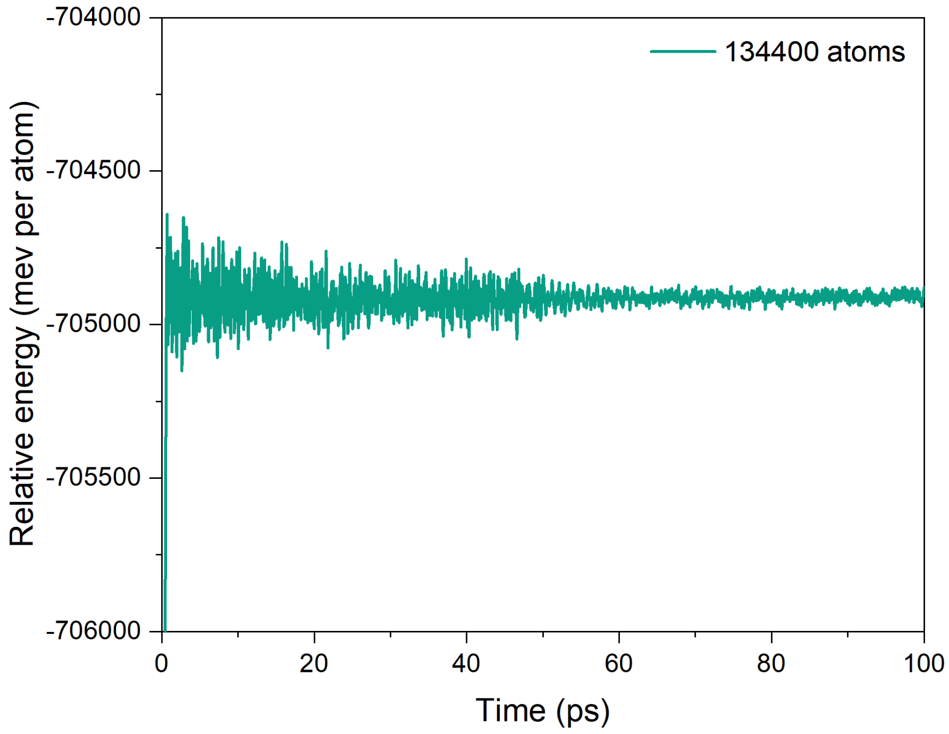

Supplementary Fig. 31 | Evolution of the total energy obtained by MLP-MD simulations for CALF-20 supercell (134,400 atoms) MLP-MD simulations in the *NVT* ensemble (T=298.15 K) and a timestep of 0.5 fs.

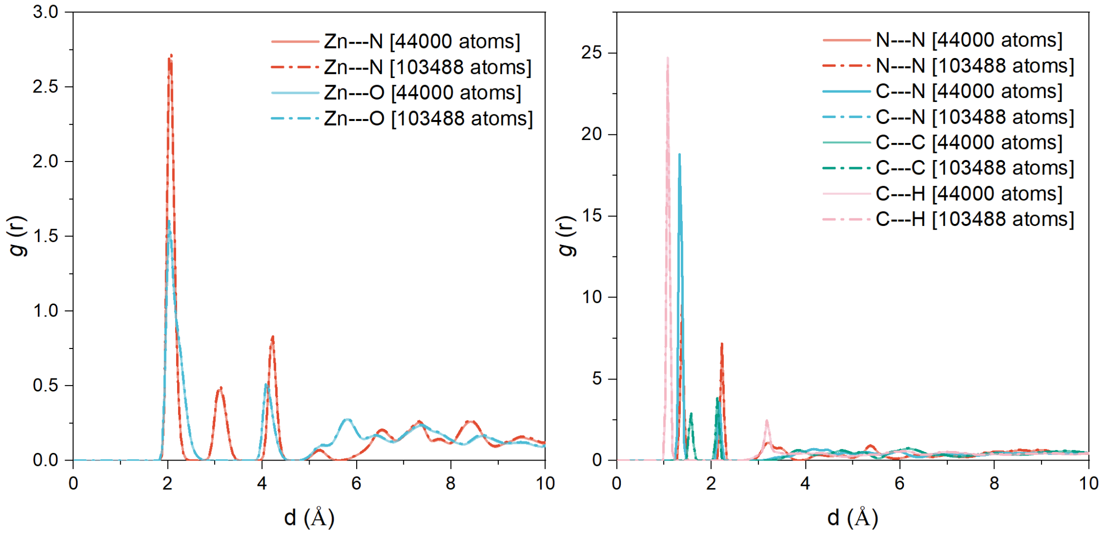

Supplementary Fig. 32 | Statistical Radial Distribution Functions (RDFs) calculated for the pairs of the different CALF-20 supercells (with 44,000 and 103,488 atoms) at room temperature by using the MLP-MD simulations.

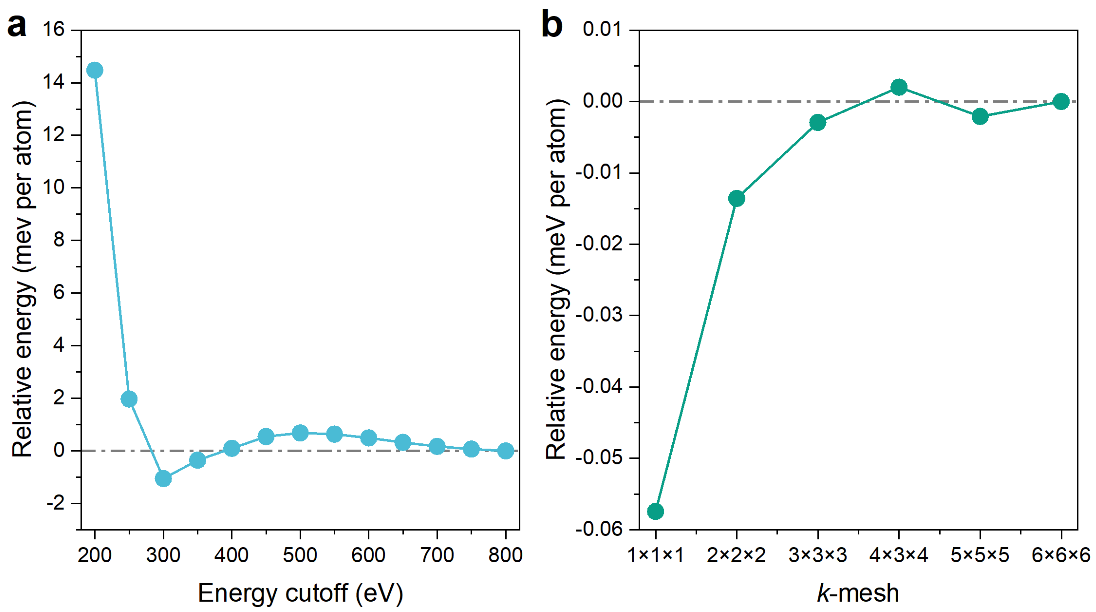

Supplementary Fig. 33 | Convergence testing of the (**a**) cut-off energy and (**b**) k-point of the CALF-20 unit-cell in the VASP code. The final choice of cut-off energy and k-points are 650 eV and 4×3×4, respectively.

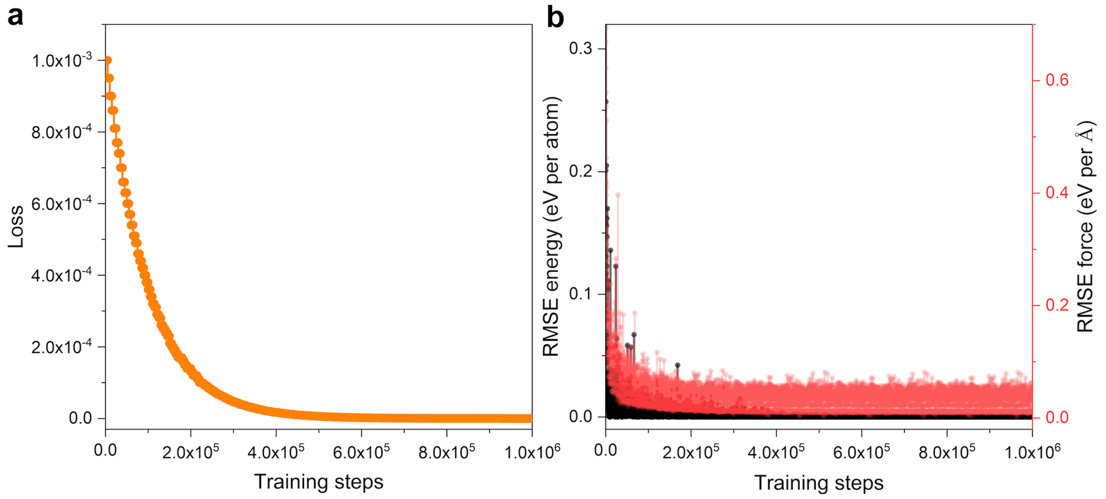

Supplementary Fig. 34 | (a) Training loss with the steps. (**b**) Variation of RMSE of force and energy with training steps.

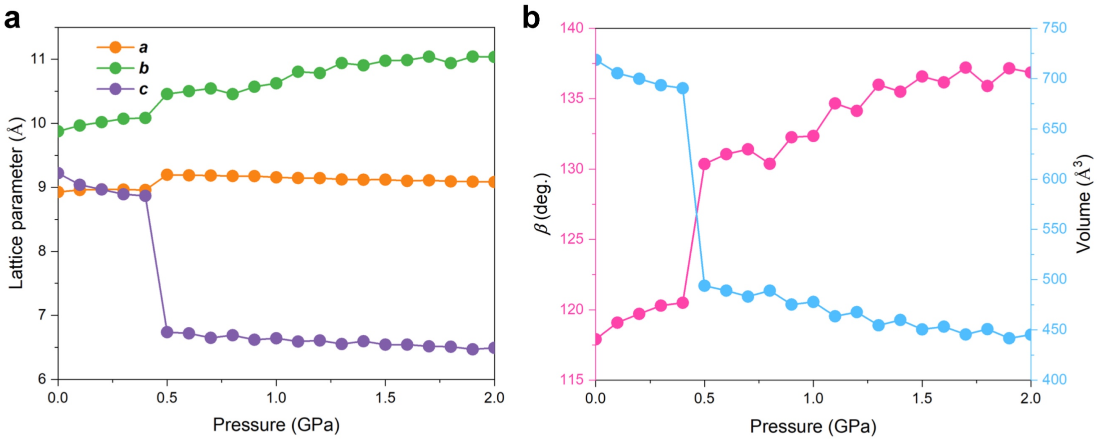

Supplementary Fig. 35 | (a, b) Unit cell parameters change of the CALF-20 during hydrostatic compression based on SCAN-D3 functional calculations.

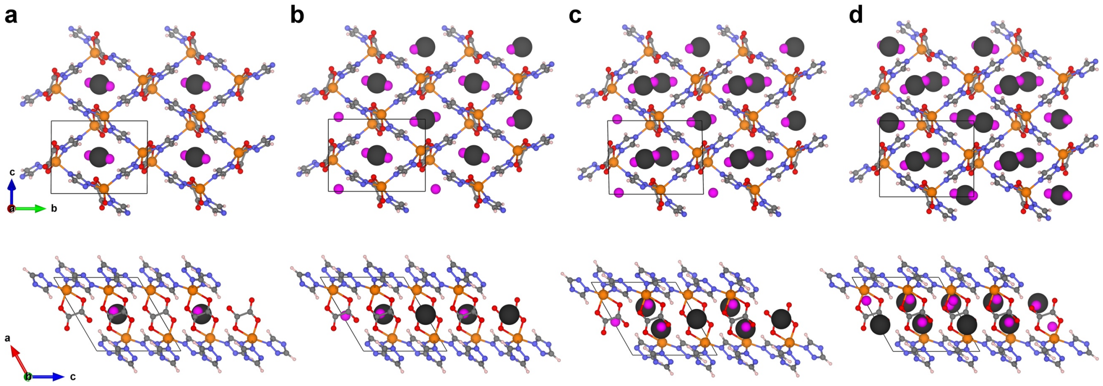

Supplementary Fig. 36 | Structural evolution of the CALF-20 unit-cell computed by DFT (using PBE-D3 functional) at different CO_2 loading (**a**, one CO_2 per unit-cell; **b**, two CO_2 per unit-cell; **c**, three CO_2 per unit-cell; **d**, four CO_2 per unit-cell).

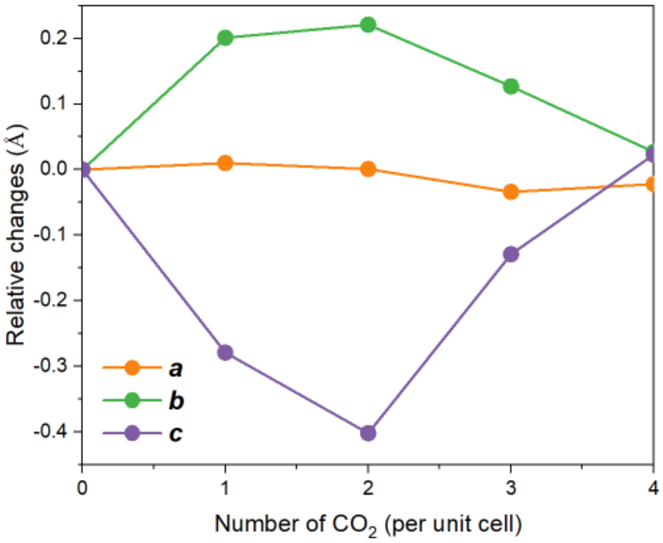

Supplementary Fig. 37 | Relative unit-cell parameters changes with different CO_2 loading of CALF-20 structure based on PBE-D3 functional calculations.

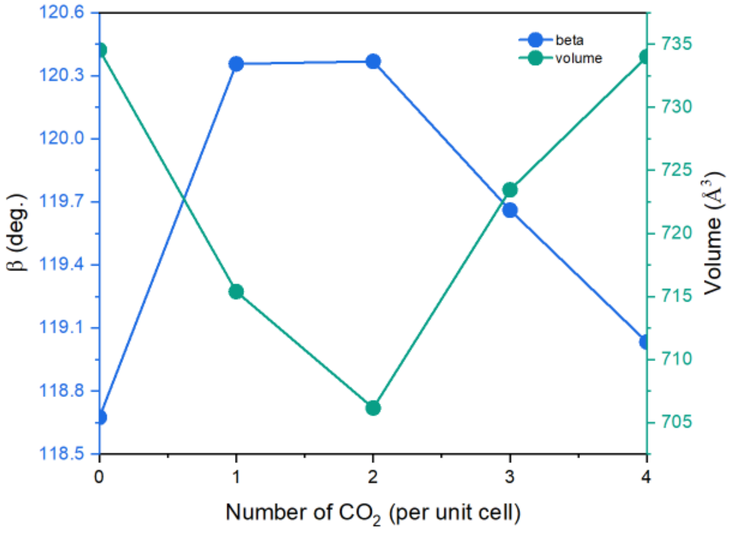

Supplementary Fig. 38 | Unit cell parameters (*β* angle and unit-cell volume) changes with different CO_2 loading of CALF-20 structure based on PBE-D3 functional calculations.

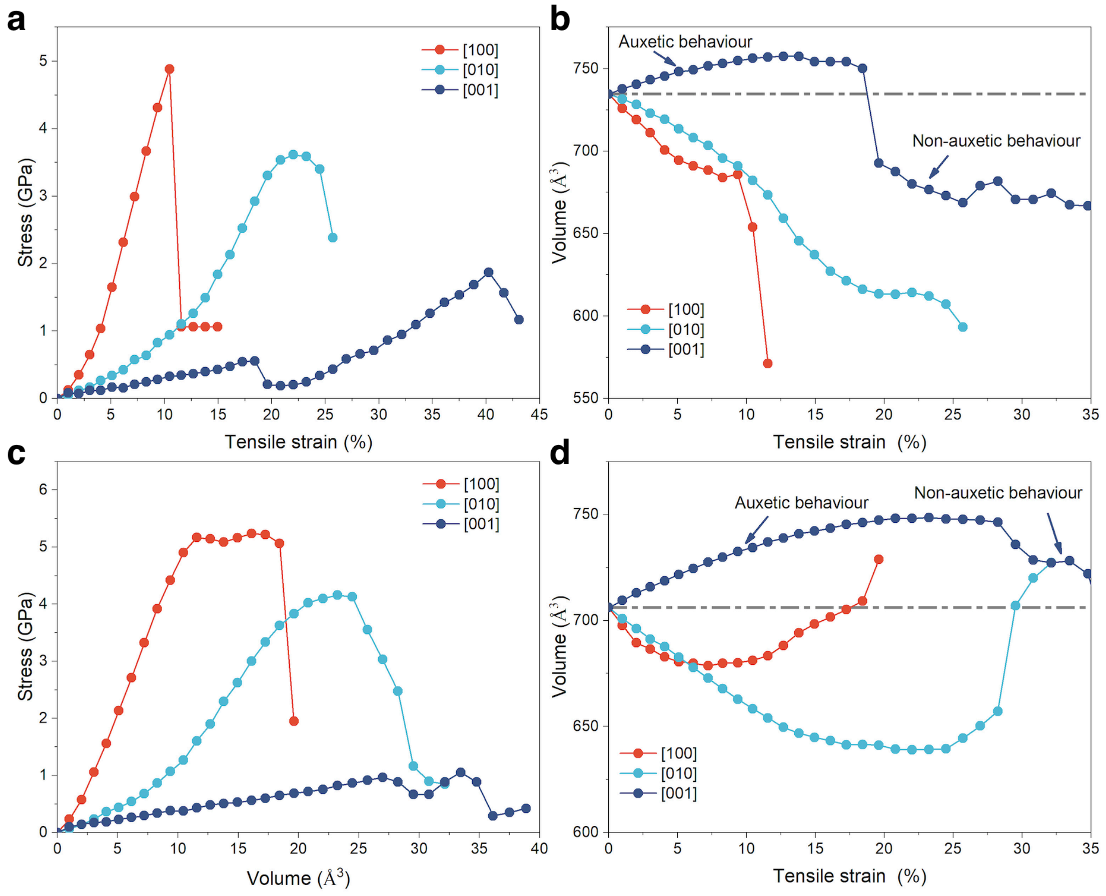

Supplementary Fig. 39 | (a) Tensile strain-stress response and (**b**) unit-cell volume changes of the pristine CALF-20 during the application of tensile strain along the three directions from DFT calculations at 0K. (**c**) Tensile strain-stress response and (**d**) unit-cell volume changes of the CALF-20 with two CO_2 loading during the application of tensile strain along the three directions from DFT calculations at 0K.

Supplementary Table 1 | Principal compressibility and corresponding principal axes determined for CALF-20.

Pressure	Principle Axes	$\boldsymbol{K_i}$ (TPa^{-1})	Direction		
			a	***b***	***c***
0~1 GPa	X_1	501.53	0.44	−0.00	0.90
	X_2	−188.77	1.00	−0.00	−0.05
	X_3	−75.52	0.00	1.00	−0.00

Supplementary Table 2 | The isotropic aggregate properties of CALF-20 by using the Voigt approach. The measurements are given in units of GPa, except for *v*, which is a dimensionless. All outcomes are at 298.15 K except for the DFT result, which is calculated at 0 K. Here UFF is the standard one and UFF-modified means modified version as reported in our previous work.[2] Data are averaged with the box deformation of 1-5% as it is in the elastic regime (except for the DFT result).

	UFF	UFF-modified	MLP	DFT
**Bulk Modulus (GPA)**	44.49 ± 0.48	45.72 ± 1.44	22.58 ± 2.32	29.13
**Young Modulus (GPA)**	82.63 ± 0.70	56.9 ± 0.53	12.99 ± 1.88	17.21
**Poisson Ratio**	0.19 ± 0.00	0.29 ± 0.00	0.40 ± 0.02	0.40
**Shear Modulus 1 (GPA)**	32.70 ± 0.29	22.44 ± 0.70	5.53 ± 1.94	6.14
**Shear Modulus 2 (GPA)**	34.76 ± 0.45	22.00 ± 0.41	4.65 ± 0.73	-

Supplementary Table 3 | Principal coefficients of thermal expansion and corresponding principal axes determined for CALF-20.

**Temperature**	**Principle Axes**	$\boldsymbol{\alpha_i}$ **(MK^{-1})**	**Direction**		
			a	***b***	***c***
10~400 K	X_1	−60.81	−0.00	1.00	−0.00
	X_2	−4.22	−0.48	0.00	0.88
	X_3	76.57	0.80	0.00	0.61

Supplementary Table 4 | Equilibrium structural parameters of the metastable CALF-20 and *β*-CALF-20.[a]

	a [Å]	***b*** [Å]	***c*** [Å]	***α*** [deg.]	***β*** [deg.]	***γ*** [deg.]	***V*** [$Å^3$]
**Exp.**	9.279	7.934	10.039	90.000	109.891	90.000	694.972
**PBE-D3**	9.297	8.062	9.901	90.000	109.848	90.000	698.020

[a] The metastable CALF-20 structure was referred to as β-CALF-20.[3]

[b] The experimental parameters are extracted from ref [3]

Supplementary Table 5 | Equilibrium structural parameters obtained by the different functionals (including [4–9]).

	a	*b*	*c*	*alpha*	*beta*	*gamma*	*V*	*Zn-O*	*Zn-N*
**Exp.**[a]	8.9138	9.6935	9.4836	90	115.8950	90	737.16	2.022/2.189	2.007/2.016/2.091
**PBE-D3**	9.0461	10.0249	9.2321	90	118.6755	90	734.54	2.028/2.243	2.012/2.033/2.110
**PBE-D3(BJ)**	9.0455	9.99266	9.23850	90	118.6014	90	733.15	2.029/2.236	2.010/2.029/2.109
**PBEsol-D3**	8.9133	9.9097	9.1897	90	118.7029	90	711.97	2.004/2.213	2.000/1.982/2.074
**revPBE-D3**	9.1263	10.1900	9.1845	90	120.0357	90	739.43	2.032/2.307	2.020/2.046/2.114
**SCAN-D3**	8.9281	9.8768	9.2219	90	117.9045	90	718.65	2.008/2.186	1.990/2.008/2.085
**SCAN-rVV10**	8.9241	9.8657	9.2286	90	117.8729	90	718.26	2.009/2.183	1.990/2.006/2.084
**SCAN-D3(BJ)**	8.9512	9.83075	9.21231	90	117.8031	90	717.08	2.008/2.181	1.992/2.007/2.085

[a] The experimental parameters are extracted from ref [1],

Supplementary Table 6 | Data preparation for the MLP training of the CALF-20 framework.

AIMD runs	Supercell	Number of snapshots
**pristine (100-800 K)**	2×2×2	14536
**1% isotropic compression (300-500 K)**	2×2×2	4821
**1% isotropic stretch (300-500 K)**	2×2×2	4713
**tensile strain (300K, including 3 directions)**	2×2×2	10000
**Total**		34070

Supplementary Table 7 | Summary of the mechanical property calculations of CALF-20 using various force fields with different deformations.

	Deformation (%)	UFF	UFF-modified	MLP-MD
**Bulk Modulus (GPA)**	**1**	45.02	47.35	20.32
	**2**	44.36	44.62	22.47
	**3**	44.08	45.2	24.97
	**4**	44.13	45.8	20.53
	**5**	44.53	45.24	19.99
**Young Modulus (GPA)**	**1**	83.7372	56.82	10.93
	**2**	82.5096	56.2212	14.18
	**3**	81.9888	56.952	12.58
	**4**	82.0818	57.708	11.70
	**5**	82.8258	57.0024	15.57
**Poisson Ratio**	**1**	0.19	0.3	0.41
	**2**	0.19	0.29	0.39
	**3**	0.19	0.29	0.42
	**4**	0.19	0.29	0.40
	**5**	0.19	0.29	0.37
**Shear Modulus 1**	**1**	32.94	22.76	2.08
	**2**	32.38	21.83	6.53

**(GPA)**		**3**	32.39	21.56	6.30
		**4**	32.86	22.95	6.23
		**5**	32.95	23.11	6.52
		**1**	34.86	21.55	3.88
		**2**	35.23	21.69	5.08
**Shear Modulus (GPA)**	**2**	**3**	34.06	22.4	4.44
		**4**	34.61	21.91	4.16
		**5**	35.02	22.46	5.68

Supplementary Note 1

MLP development of the CALF-20 structure.

It is well known that DFT is computationally feasible for the mechanical properties of periodic materials, however, the limitation of DFT calculation is that it's challenging to handle the properties at finite temperatures, especially for MOF systems which usually have large length scales. Therefore, in this section, we mainly focus on the development of the machine learning potential (MLP) for CALF-20 in order to obtain a high-quality MLP for subsequent investigation at ambient pressure and temperature that comparable to experimental investigations. Supplementary Fig. 5 compare the energies and forces of the MLP with the values of the CALF-20 based on the DFT calculations. It can be seen that both energy and force are in excellent agreement. The root-mean square error of the energy and force are 0.068 meV atom^{-1} and 0.023 eV Å^{-1}, respectively, indicating the high accuracy of the developed MLP in describing the features of the learned system. As shown in Supplementary Fig. 6, the calculated volume-energy relationship also demonstrates that the well-trained MLP successfully describes the energy of the deformation phases of the CALF-20 compared to DFT outputs, suggesting that the development MLP can be efficiently applied to the CALF-20 with variable lattice deformation (*cf.* Supplementary Fig. 7 for the comparison of total energy calculated from DFT and MLP for CALF-20 at different pressures). More impressively, MLP describes the phonons of CALF-20 accurately, as show in Supplementary Fig. 8a and Supplementary Fig. 8b. According to our MLP-based calculations, the computation yields three acoustic and 129 optical branches, where the acoustic branches only exist in the low frequency range. The absence of any imaginary frequencies in the phonon dispersion indicates the dynamical stability of CALF-20. A distinct feature is the considerable phonon softening of the transverse acoustic branch along the wavevector (*e.g.* B−D, Y−C, see Supplementary Fig. 8a and Brillouin zone path in Supplementary Fig. 9), which generally leads to negative thermal expansion also (See discussion in the main text for more information).[10] The highest vibrational mode peak at 96.2 THz (~3209 cm^{-1}) belongs to the stretching mode of the C-H bond,

while the metal-node vibrational mode is predominantly located in the low frequency regime (below 10 THz), as shown in Supplementary Fig. 8b and Supplementary Fig. 10. It should be emphasized here that it is very time-consuming to compute the phonon properties of CALF-20 (44 atoms in unit cell) by DFT calculations owing to the large unit cell, while our results demonstrate that the MLP method can obtain accurate lattice vibrational information, especially for a large system such as MOF, where its advantages are particularly pronounced (*cf.* Supplementary Fig. 11 and Supplementary Note 2 for details).

We further explore the capability of the trained MLP in describing the structural dynamics of the lattice at finite temperatures (300-500 K). Supplementary Fig. 12 and Supplementary Fig. 13 illustrate the total energy oscillation of the CALF-20 *versus* simulation time (up to 1 nanosecond [ns]) and the comparison of the radial distribution function (RDF) of different pairs in CALF-20 in the AIMD and MLP-MD simulations. Clearly, the structure attains energetic equilibrium after 0.1 ns, and the RDF curves from MLP-MD and AIMD are in good agreement, demonstrating the reliability of the MLP in the description of the CALF-20 structure at finite temperatures. It was also discovered that the atoms in the structure remained undiffused even at 500 K, demonstrating the high-temperature stability of the CALF-20 structure. (Supplementary Fig. 14) Therefore, the above-mentioned protocols show that the MLP technique accurately predicts the flexibility of CALF-20 at different temperatures. It maintains the precision of DFT level and ensures robust simulation outcomes from MLP-MD simulations.

Supplementary Note 2

Performance and transferability of the MLP for huge systems of CALF-20

It is well-recognized that the AIMD simulation is challenging with thousands of atoms, and scaling it to the nanosecond scale is even more unrealistic. However, the previous results already show that it is possible to obtain results with high-accuracy while significantly accelerating the simulation speed using MLP approaches.[11–13] As shown in Supplementary Fig. 11, AIMD scales in general as $\sim O(N^3)$, with N being the number of atoms, as can be expected. However, the MLP-MD scaling is roughly as $\sim O(N^1)$, implying that the simulation time increases linearly with the size of the systems, rather than exponentially. This improvement in efficiency is significant for MOF simulations, as the unit cells of MOFs are typically large, and it would be very expensive to use conventional AIMD simulations.

As shown in Supplementary Fig. 28 and Supplementary Fig. 29, we tested two different large-scale systems (with 44,000 and 134,400 atoms, respectively), and such a system is not feasible with any AIMD simulations. We carried out MLP-MD simulations using NVT ensemble (Nosé thermostat[14]) for 100 ps simulation (0.5 fs time step) at room temperature. It can be seen that the structures remain stable throughout the simulation process (Supplementary Fig. 30 and Supplementary Fig. 31). The structural evolution during the simulation is represented by animations in Supplementary Movie 3 and Supplementary Movie 4. Even in the system of more than 100,000 atoms, no structure collapse was found during the simulation process, proving that our MLP is very well-trained. At the same time, the RDFs obtained by different sizes are almost the same, as shown in the Supplementary Fig. 32, which demonstrates a good transferability of the trained MLP.

Supplementary Note 3

DFT calculations

The DFT calculation was performed by using the Vienna *ab initio* simulation package (VASP, version: 5.4.4)[15] with the projector augmented waves (PAW) method and the Perdew-Burke-Ernzerhof (PBE) exchange-correlation functional.[4,16] The electron configurations of the pseudopotential are chosen as Zn: $d^{10}p^2$, N: s^2p^3, O: s^2p^4, C: s^2p^2 and H: s^1. The plane wave energy cutoff was set at 650 eV (ENCUT=650). We used a Davidson block iteration scheme (IALGO=38) for electronic energy minimization. Brillouin zone integrations were conducted in *k*-space (LREAL=.FALSE.) using a Γ-centered 4×3×4 Monkhorst-Pack mesh. Accelerated convergence of the self-consistent calculations was achieved using a modified Broyden method.[17] Both atom positions and unit cell parameters were fully relaxed during the structure optimization (ISIF=3), allowing symmetry breaking (ISYM = 0). The ionic relaxation steps were performed using the conjugate-gradient method (IBRION=2). The convergence criteria for energy and force in structural optimization were set to 10^{-5} eV and 10^{-3} eV/Å, respectively. The van der Waals interaction corrections were also considered by using DFT-D3 corrections.[5]

The elastic moduli were calculated using the finite difference approach as implemented in the VASP code.[15] The stiffness tensor is determined by performing six finite distortions of the lattice.[18] Since high accuracy is required to determine the elastic constants,[19] the convergence of the strain energies with respect to the Brillouin zone integration has been carefully checked. Based on our tests, we selected the 650 eV for and the 6×6×6 *k*-mesh, as shown in the Supplementary Fig. 33. In addition, we used very high convergence criteria for energy (EDIFF = 0.1E-07) and force (EDIFFG = -.2E-04) to calculate the elastic moduli accurately. All calculations were performed in reciprocal space. (LREAL=.FALSE.)

Commencing from the fully relaxed CALF-20 structure, we systematically apply uniaxial tensile strain, progressively reaching up to 45% with ~1% increments. The definition of tensile strain is expressed as:

$$\varepsilon = \frac{a - a_0}{a_0} \quad (1)$$

wherein a and a_0 denote the lattice constants of the strained and relaxed structures, respectively. When strain is introduced in a specific direction, the lattice constant in the perpendicular direction is relaxed completely by minimizing the total energy and force, ensuring the absence of stress in that perpendicular direction. This approach is implemented through the process of recompiling the VASP package.[15] All DFT computations, including structural optimization and molecular dynamics simulations, were performed by using VASP package[15] using high-precision setting (PREC=.ACCURATE.). The bonding of the selected atoms in CALF-20 was further characterized by the Integrated Crystal Orbital Hamiltonian Populations (ICOHP) using the LOBSTER package.[20]

Thermal expansion based quasi-harmonic approximation method

The thermal and mechanical properties of CALF-20 were also calculated by using the quasi-harmonic approximation (QHA) approach as carried out in Phonopy code,[21] that incorporating the volume dependence of phonon frequencies as an integral part of the anharmonic effects.[22] First, a sequence of phonon spectra was computed using varying lattice constants. This was achieved by adjusting the lattice's scaling factor, with a fixed scaling rate of 1%. The analysis encompassed both volume contraction and expansion, with 5 data points allocated to each scenario (11 data point finally including the pristine CALF-20, as shown in Supplementary Fig. 15). For each lattice constant a, the free energy can be obtained from:

$$F(a,T) = G_a + \sum_{q,j} \frac{\hbar\omega_{a;q,j}}{2} + \frac{1}{\beta}\sum_{q,j} \ln\left[1 - \exp\left(-\beta\hbar\omega_{a;q,j}\right)\right] \quad (2)$$

where G_a is the ground-state free energy; $\hbar$ is the reduced Planck constant; $\omega_{a;q,j}$ means the vibrational frequency to the corresponding vector q of j; $\hbar$ is the reduced Planck constant; $\beta = \frac{1}{Tk_B}$ where T means temperature and k_B is Boltzmann constant; The Birch-Murnaghan equation of state is used to fit the data (energy-volume data). Then the equilibrium lattice constants at different temperature is obtained. Finally, the thermal expansion coefficient at different temperature can be calculated from following:

$$\delta_T = \frac{\partial \ln a(T)}{\partial T} \quad (3)$$

Thermal expansion and compression of CALF-20 obtained by empirical expression

The principal coefficients of thermal expansion and compressibility from variable-temperature and variable-pressure lattice parameter data were calculated based on *PASCal* (principal axis strain calculator) code.[23] For the compression computations, the data of lattice parameters changes with the pressure calculated by DFT using PBE-D3 functional were adopted. For the thermal expansion, the MLP-MD approach was chosen because it is almost impossible to achieve the equilibrium lattice parameters at finite temperature by DFT. A 2 × 2 × 2 supercell was employed during the MLP-MD simulation using *NPT* ensemble at 1 bar pressure, and the total simulation time reached 1 ns. Finally, we evaluated the lattice information of the last 500 ps to calculate the thermal expansion. The specific calculation follows the equation:

$$x_P = x_0 + \kappa|P - P_C|^{\lambda} \quad (4)$$

$$x_T = x_0 + \kappa T|\frac{T}{T - T_C}|^{\lambda} \quad (5)$$

ML dataset preparation

The accuracy and transferability of the machine-learning potential (MLPs) are highly sensitive to the availability of atomic structure databases covering the relevant regions of the configurations space. Specifically, our target is to develop a MLP that can accurately describe the flexibility of the CALF-20, a comprehensive sampling of the potential energy surface (PES) is necessary. For this reason, we primarily use the AIMD simulations to increase the diversity to the maximum extent possible in order to cover the PES of the structure. We consider the structural deformation caused by isotropic pressure and isotropic tension, as well as the structural deformation of structures in different directions under tensile strain conditions. At the same time, for each scenario, we consider the structural perturbations caused by different temperatures (100 K~800 K). Detailed information on the species and number of the training structures in our dataset can be found in Supplementary Table 6.

All AIMD simulations were performed by using the VASP package with the canonical (*NVT*) ensemble with the Nosé thermostat[14] and removing symmetry

constraints during the simulations (ISYM=0). The GGA-PBE approximation was employed for the exchange correlation functional and the DFT-D3 method of Grimme was used to consider the dispersion corrections.[5] The 650 eV energy cutoff was used and a gamma point was used to sample the Brillouin zone. All the simulations were performed in a 2 × 2 × 2 supercell. It should be noted that in order to increase the structural sampling, we performed AIMD simulations of the configurations of the CALF-20 supercell structure in the different conditions as shown in Supplementary Table 6. The simulation time for each AIMD simulation was around 1-2 ps which depends on the system.

MLP training and validation

The DeePMD-kit package[24] (version 2.0.1) was employed to train MLPs for predicting energies, forces, and stress tensors based on the training data associated with CALF-20 systems. In the such approach, a first network of descriptors was used to transform the local atomic environments into a series of embeddings that maintained translation, rotation and permutation invariances. A second network then utilized these embeddings to make predictions regarding the energy, forces, and virial of the system. For this purpose, the "*se_2_a*" descriptor from DeepMD, which contains both radial and angular information, was adopted. Both networks utilize the ResNet architecture.[25] The DeepPot-SE model was used during the training process.[26] The size of the fitting network was set to {240, 240, 240}. The cutoff radius was set as 7.9 Å with the smoothing parameter of 2.1 Å. The batch and step decay rate parameters were configured with values of 1,000,000 and 5,000, respectively. Initially, the learning rate was established at 0.001 and was scheduled to decrease every 5,000 steps. The total number of batches was set to 1,000,000.

During the MLP training, we tracked the RMSE (root mean squared error) value as it decreased exponentially. Supplementary Fig. 34 shows the loss function and RMSE values as they evolved during training for CALF-20. It's important to note that due to the features of the DFT data and various factors affecting network performance, achieving zero RMSE for force and energy is extremely difficult. Nevertheless, our

results indicate that the trained RMSE results can achieve a level of accuracy consistent with the DFT scale.

MLP-MD simulations

The MLP derived from the DFT was applied in the following MLP-MD simulations through integration with the DeepMD-kit interface coupled with the LAMMPS code.[24,27] In the MLP-MD simulations, the trained model served as a pair style within the LAMMPS framework, enabling the computation of both energy and force profiles during the MD simulations. The phonon dispersion spectra were calculated under strict convergence criteria with the Phonopy code,[21] by using LAMMPS as the calculator.[28]

Classical MD simulations

The force-field parameters as well as the atomic partial charges for CALF-20 were taken from UFF[29] and DDEC06[30] partial charges, respectively. The flexibility of the structure was considered in whole simulation process. The Lorentz−Berthelot mixing rules[31] were applied to compute the cross 12−6 LJ parameters. The cut-off for LJ interactions was 12 Å, and electrostatic interactions were calculated via the Ewald summation method[32] with a tolerance of 10^{-6}. Temperature and pressure were regulated by Nosé-Hoover thermostat[14] and barostat respectively. Coupling constant of both thermo- and barostat was 0.1 ps. Time step of the simulation was chosen to be 0.5 fs. These MD simulations were performed using the LAMMPS simulation software.[27]

Mechanical properties from classical MD and MLP-MD at finite temperature

The mechanical properties, namely Young Modulus, Bulk Modulus, and Poisson's ratio of the MOF, are calculated using the explicit deformation method as implemented in the LAMMPS simulation software. The underlying theory of the methodology is as follows:

The elastic stiffness tensor in the small deformation regime can be written by the generalized Hook's law as

$$\sigma_{\alpha\beta} = C_{\alpha\beta\mu\nu}\epsilon_{\mu\nu} \qquad (6)$$

where σ is the stress tensor, C is a rank four stiffness tensor, ϵ is the strain tensor and α, β, μ, ν represent the Cartesian coordinates. It can be shown that ϵ and σ are symmetric tensors and can be written as a vector with six independent components and C as a six-by-six matrix. The generalized Hooke's law is then written as:

$$\sigma_i = C_{ij}\epsilon_j \quad (7)$$

In MD simulations, the instantaneous stress tensor is computed using the virial theorem, which takes the following form:

$$\sigma_{\alpha\beta} = -\frac{1}{V}\left[\sum_{i=1}^{N} m_i v_{\alpha,i} v_{\beta,i} + \sum_{i=1}^{N} \sum_{j>i} \left(-\frac{\partial u(r)}{\partial r}\right)_{r=r_{ij}} \frac{r_{\alpha,ij} r_{\beta,ij}}{\|r_{ij}\|}\right] \quad (8)$$

where V is the volume of the simulation box, N is the number of particles in the simulation box, $u(r)$ is the pair potential, m_i, v_i, r_i is the mass, velocity, and position of particle i, respectively and r_{ij} is the distance between the particle i and j.

In this work, we used six different deformation types and extracted C_{ij} from the slope of the corresponding stress–strain graph. In the first three deformations, the box is elongated either in x- or y- or z-direction by a small amount ξ. During this deformation at a particular direction (for this case, x-direction), each point in the periodic cell is transformed as follows:

$$(x\, y\, z) = (x(1+\xi)\, y\, z) \quad (9)$$

and the strain tensor is, $\epsilon = (\xi, 0, 0, 0, 0, 0)$. During the last three deformations, the box is deformed into a monoclinic box by changing one of the angles at constant volume. During this deformation at a particular direction (for this case, along angle a-vector by an angle φ), each point in the periodic cell is transformed as follows:

$$(x\, y\, z) = (x\, y\, z + z tan(\varphi\xi)) \quad (10)$$

and the strain tensor is, $\epsilon = (0, 0, 0, tan(\varphi\xi), 0, 0)$.

For each deformation, we run 1 ns of equilibration and 1 ns of production at 300 K for each of the six directions in both positive and negative axis, totaling 24 ns of simulation. Five elementary strains in the range of 1-5% extension of the material have been conducted to calculate the Young Modulus, Bulk Modulus, Shear Modulus and Poisson's ratio. (Supplementary Table 7)

Supplementary Note 4

The reason for selecting the PBE-D3 functional

There are many different functionals to consider in DFT calculations. We have tested a few commonly used functionals and also considered different dispersion correction schemes. As can be seen in Supplementary Table 5, we found that for all the PBE-based functionals tested, with the exception of PBEsol-D3 which seriously underestimates the volume of the unit cell, the parameters of the CALF-20 structure can be predicted reasonably well compared to the experiment. We finally chose PBE-D3 functional for the DFT calculations. (Unless otherwise stated, all results are based on PBE-D3)

Supplementary Note 5

Effect of CO_2 adsorption on the structure of CALF-20

DFT geometry optimization of the MOF structure loaded with different CO_2 concentrations up to the experimental saturation reported in the initial paper[1] (1~4 CO_2 molecules per unit cell) were carried at the same level of theory than used for the exploration of the MOF structure (PBE-D3) (Supplementary Fig. 36, Supplementary Fig. 37, and Supplementary Fig. 38). We observed that when CO_2 loading increases, the lattice parameters of the CALF-20 show anisotropic evolution, arising from the anisotropic mechanical properties of the CALF-20 structure (see main text discussion). In particular, the ***c***-parameter decreases the greatest when two CO_2 molecules per unit cell are loaded in the pores of CALF-20, and the unit cell volume decreases by 3.9% (*cf.* Supplementary Fig. 37 and Supplementary Fig. 38). The primary origin of this contraction is that the confined CO_2 molecules at this loading occupy the central position of the pore of the CALF-20 structure, resulting in the internal chemical pressure generating the most pronounced volume shrinkage.

We next explored the effect of CO_2 for a given loading of 2 CO_2 per unit-cell on the stress-strain behaviour of the CALF-20 structure. As shown in Supplementary Fig. 39, the introduction of CO_2 significantly improves the ductility of the CALF-20 structure, particularly along the [001] direction. The failure strain point increased from 18.4% (strain-softening point) to 26.9%, but simultaneously, we revealed that the presence of CO_2 prevents the formation of the strain-induced metastable phase evidenced for the empty case. Interestingly we reveal that the introduction of CO_2 also increases the auxetic region of the CALF-20 structure, and the structure retains its auxetic regime under a tensile strain of 23%.

Supplementary References

1. Lin, J.-B. *et al.* A scalable metal-organic framework as a durable physisorbent for carbon dioxide capture. *Science* **374**, 1464–1469 (2021).
2. Naskar, S., Fan, D., Ghoufi, A. & Maurin, G. Microscopic insight into the shaping of MOFs and its impact on CO 2capture performance. *Chemical Science* **14**, 10435–10445 (2023).
3. Chen, Z. *et al.* Humidity-Responsive Polymorphism in CALF-20: A Resilient MOF Physisorbent for CO_2 Capture. *ACS Materials Lett.* 2942–2947 (2023) doi:10.1021/acsmaterialslett.3c00930.
4. Perdew, J. P., Burke, K. & Ernzerhof, M. Generalized Gradient Approximation Made Simple. *Phys. Rev. Lett.* **77**, 3865–3868 (1996).
5. Grimme, S. Semiempirical GGA-type density functional constructed with a long-range dispersion correction. *J. Comput. Chem.* **27**, 1787–1799 (2006).
6. Perdew, J. P. *et al.* Restoring the Density-Gradient Expansion for Exchange in Solids and Surfaces. *Phys. Rev. Lett.* **100**, 136406 (2008).
7. Zhang, Y. & Yang, W. Comment on "Generalized Gradient Approximation Made Simple". *Phys. Rev. Lett.* **80**, 890–890 (1998).
8. Sun, J., Ruzsinszky, A. & Perdew, J. P. Strongly Constrained and Appropriately Normed Semilocal Density Functional. *Phys. Rev. Lett.* **115**, 036402 (2015).
9. Sun, J. *et al.* Accurate first-principles structures and energies of diversely bonded systems from an efficient density functional. *Nature Chem* **8**, 831–836 (2016).

10. Zhou, W., Wu, H., Yildirim, T., Simpson, J. R. & Walker, A. R. H. Origin of the exceptional negative thermal expansion in metal-organic framework-5 Zn 4 O (1 , 4 − benzenedicarboxylate) 3. *Phys. Rev. B* **78**, 054114 (2008).

11. Jinnouchi, R., Karsai, F. & Kresse, G. On-the-fly machine learning force field generation: Application to melting points. *Phys. Rev. B* **100**, 014105 (2019).

12. Deringer, V. L., Caro, M. A. & Csányi, G. A general-purpose machine-learning force field for bulk and nanostructured phosphorus. *Nat Commun* **11**, 5461 (2020).

13. Pinheiro, M., Ge, F., Ferré, N., Dral, P. O. & Barbatti, M. Choosing the right molecular machine learning potential. *Chem. Sci.* **12**, 14396–14413 (2021).

14. Nosé, S. A unified formulation of the constant temperature molecular dynamics methods. *The Journal of Chemical Physics* **81**, 511–519 (1984).

15. Kresse, G. & Furthmüller, J. Efficient iterative schemes for *ab initio* total-energy calculations using a plane-wave basis set. *Phys. Rev. B* **54**, 11169–11186 (1996).

16. Blöchl, P. E. Projector augmented-wave method. *Phys. Rev. B* **50**, 17953–17979 (1994).

17. Johnson, D. D. Modified Broyden's method for accelerating convergence in self-consistent calculations. *Phys. Rev. B* **38**, 12807–12813 (1988).

18. Le Page, Y. & Saxe, P. Symmetry-general least-squares extraction of elastic data for strained materials from *ab initio* calculations of stress. *Phys. Rev. B* **65**, 104104 (2002).

19. Caro, M. A., Schulz, S. & O'Reilly, E. P. Comparison of stress and total energy methods for calculation of elastic properties of semiconductors. *J. Phys.: Condens. Matter* **25**, 025803 (2013).
20. Maintz, S., Deringer, V. L., Tchougréeff, A. L. & Dronskowski, R. LOBSTER: A tool to extract chemical bonding from plane-wave based DFT: Tool to Extract Chemical Bonding. *J. Comput. Chem.* **37**, 1030–1035 (2016).
21. Togo, A. & Tanaka, I. First principles phonon calculations in materials science. *Scripta Materialia* **108**, 1–5 (2015).
22. Togo, A., Chaput, L., Tadano, T. & Tanaka, I. Implementation strategies in phonopy and phono3py. *J. Phys.: Condens. Matter* **35**, 353001 (2023).
23. Cliffe, M. J. & Goodwin, A. L. *PASCal* : a principal axis strain calculator for thermal expansion and compressibility determination. *J Appl Crystallogr* **45**, 1321–1329 (2012).
24. Wang, H., Zhang, L., Han, J. & E, W. DeePMD-kit: A deep learning package for many-body potential energy representation and molecular dynamics. *Computer Physics Communications* **228**, 178–184 (2018).
25. He, K., Zhang, X., Ren, S. & Sun, J. Deep Residual Learning for Image Recognition. *Proceedings of the IEEE Conference on Computer Vision and Pattern Recognition (CVPR)* 770–778 (2016).
26. Zhang, L., Han, J., Wang, H., Car, R. & E, W. Deep Potential Molecular Dynamics: A Scalable Model with the Accuracy of Quantum Mechanics. *Phys. Rev. Lett.* **120**, 143001 (2018).

27. Thompson, A. P. *et al.* LAMMPS - a flexible simulation tool for particle-based materials modeling at the atomic, meso, and continuum scales. *Computer Physics Communications* **271**, 108171 (2022).

28. Carreras, A. phonoLAMMPS: a python interface for LAMMPS phonon calculations using phonopy. *Zenodo* (2020) doi:https://doi.org/10.5281/zenodo.3940626.

29. Rappe, A. K., Casewit, C. J., Colwell, K. S., Goddard, W. A. & Skiff, W. M. UFF, a full periodic table force field for molecular mechanics and molecular dynamics simulations. *J. Am. Chem. Soc.* **114**, 10024–10035 (1992).

30. Manz, T. A. & Limas, N. G. Introducing DDEC6 atomic population analysis: part 1. Charge partitioning theory and methodology. *RSC Adv.* **6**, 47771–47801 (2016).

31. Lorentz, H. A. Ueber die Anwendung des Satzes vom Virial in der kinetischen Theorie der Gase. *Annalen der physik* **248**, 127–136 (1881).

32. Ewald, P. P. Die Berechnung optischer und elektrostatischer Gitterpotentiale. *Annalen der physik* **369**, 253–287 (1921).
