# Supplementary material for: Unconventional mechanical and thermal behaviours of MOF CALF-20
Source: Nat Commun. 2024 Apr 16;15:3251. doi: 10.1038/s41467-024-47695-6 (PMC11021538; doi:10.1038/s41467-024-47695-6)
Supplement: Supplementary file 3 — Description of Additional Supplementary Files [file 41467_2024_47695_MOESM3_ESM.pdf]

Description of Additional Supplementary Files

Supplementary Movie 1

Description: The structural evolution of CALF-20 supercell with 44,000 atoms during NVT MLP-MD simulation

Supplementary Movie 2

Description: The structural evolution of CALF-20 supercell with 134,400 atoms during NVT MLP-MD simulation.

Supplementary Movie 3

Description: The structural evolution of the CALF-20 by using MLP-MD simulation with tensile strain applied along the [001] direction.

Supplementary Movie 4

Description: The dynamic process of transition from pristine CALF-20 to metastable CALF-20 structure based on CINEB calculation (displayed as supercell).
